# Sex and statin-related genetic associations at the *PCSK9* gene locus: results of genome-wide association meta-analysis

**DOI:** 10.1186/s13293-024-00602-6

**Published:** 2024-03-26

**Authors:** Janne Pott, Azin Kheirkhah, Jesper R. Gadin, Marcus E. Kleber, Graciela E. Delgado, Holger Kirsten, Lukas Forer, Stefanie M. Hauck, Ralph Burkhardt, Hubert Scharnagl, Markus Loeffler, Winfried März, Joachim Thiery, Christian Gieger, Annette Peters, Angela Silveira, Ferdinand van’t Hooft, Florian Kronenberg, Markus Scholz

**Affiliations:** 1 Institute for Medical Informatics, Statistics and Epidemiology, University of Leipzig, Leipzig, Germany; 2 MRC Biostatistics Unit, University of Cambridge, Cambridge, UK; 3 Institute of Genetic Epidemiology, Medical University of Innsbruck, Innsbruck, Austria; 4 Division of Cardiovascular Medicine, Department of Medicine Solna, Karolinska Institutet, Karolinska University Hospital Solna, Solna, Sweden; 5 Vth Department of Medicine, Medical Faculty Mannheim, University of Heidelberg, Mannheim, Germany; 6 SYNLAB MVZ Humangenetik Mannheim, Mannheim, Germany; 7 LIFE Research Center for Civilization Diseases, University of Leipzig, Leipzig, Germany; 8 Metabolomics and Proteomics Core, Helmholtz Zentrum München, German Research Center for Environmental Health, Neuherberg, Germany; 9 Institute of Clinical Chemistry and Laboratory Medicine, University Hospital Regensburg, Regensburg, Germany; 10 Clinical Institute of Medical and Chemical Laboratory Diagnostics, Medical University of Graz, Graz, Austria; 11 SYNLAB Academy, Synlab Holding Deutschland GmbH, Mannheim and Augsburg, Germany; 12 Institute of Laboratory Medicine, Clinical Chemistry and Molecular Diagnostics, University Hospital Leipzig, Leipzig, Germany; 13 Faculty of Medicine, University of Kiel, Kiel, Germany; 14 Institute of Epidemiology, Helmholtz Zentrum München, German Research Center for Environmental Health, Neuherberg, Germany

**Keywords:** PCSK9, GWAS, Sex, Statin, Interaction

## Abstract

**Background:**

Proprotein convertase subtilisin/kexin type 9 (PCSK9) is a key player of lipid metabolism with higher plasma levels in women throughout their life. Statin treatment affects PCSK9 levels also showing evidence of sex-differential effects. It remains unclear whether these differences can be explained by genetics.

**Methods:**

We performed genome-wide association meta-analyses (GWAS) of PCSK9 levels stratified for sex and statin treatment in six independent studies of Europeans (8936 women/11,080 men respectively 14,825 statin-free/5191 statin-treated individuals). Loci associated in one of the strata were tested for statin- and sex-interactions considering all independent signals per locus. Independent variants at the *PCSK9* gene locus were then used in a stratified Mendelian Randomization analysis (cis-MR) of PCSK9 effects on low-density lipoprotein cholesterol (LDL-C) levels to detect differences of causal effects between the subgroups.

**Results:**

We identified 11 loci associated with PCSK9 in at least one stratified subgroup (*p* < 1.0 × 10^–6^), including the *PCSK9* gene locus and five other lipid loci: *APOB*, *TM6SF2*, *FADS1*/*FADS2*, *JMJD1C*, and *HP*/*HPR*. The interaction analysis revealed eight loci with sex- and/or statin-interactions. At the *PCSK9* gene locus, there were four independent signals, one with a significant sex-interaction showing stronger effects in men (rs693668). Regarding statin treatment, there were two significant interactions in *PCSK9* missense mutations: rs11591147 had stronger effects in statin-free individuals, and rs11583680 had stronger effects in statin-treated individuals. Besides replicating known loci, we detected two novel genome-wide significant associations: one for statin-treated individuals at 6q11.1 (within *KHDRBS2*) and one for males at 12q24.22 (near *KSR2*/*NOS1*), both with significant interactions. In the MR of PCSK9 on LDL-C, we observed significant causal estimates within all subgroups, but significantly stronger causal effects in statin-free subjects compared to statin-treated individuals.

**Conclusions:**

We performed the first double-stratified GWAS of PCSK9 levels and identified multiple biologically plausible loci with genetic interaction effects. Our results indicate that the observed sexual dimorphism of PCSK9 and its statin-related interactions have a genetic basis. Significant differences in the causal relationship between PCSK9 and LDL-C suggest sex-specific dosages of PCSK9 inhibitors.

**Supplementary Information:**

The online version contains supplementary material available at 10.1186/s13293-024-00602-6.

## Abstract (plain language summary)

The protein “proprotein convertase subtilisin/kexin type 9” (PCSK9) regulates the levels of low-density lipoprotein cholesterol (LDL-C) in blood, and thus, contributes to the risk of cardio-vascular diseases. Women tend to have higher PCSK9 plasma levels throughout their life, although the difference is smaller in patients under LDL-C lowering medication (e.g., statins). We investigated the interplay of genetics, statin-treatment and sex, using combined data from six European studies. We detected 11 genetic regions associated with PCSK9 levels, of which one was specific for women (at *SLCO1B3*, a statin-transporter gene), and three were specific for men (e.g., *ALOX5*, encoding a protein linked to chronic inflammatory diseases such as atherosclerosis). We also tested if statin use changed the genetic effect and found five genes only associated with PCSK9 levels in untreated participants. Variants in the gene encoding PCSK9 were most strongly associated and had heterogeneous effects in dependence on statin treatment and sex: On one hand, there were genetic variants with stronger effects in men than women. Those variants are also linked to sex-differential gene expression of PCSK9. On the other hand, there were also variants with treatment-depending effects, linked to protein structure and functionality of PCSK9. This indicates that the observed sexual and treatment-related effects on PCSK9 levels have a genetic basis. In addition, we compared the causal effects of PCSK9 on LDL-C levels between men and women and found a different response to statin treatment. This highlights the need for sex-sensitive dosages of lipid-lowering medication.

**Supplementary Information:**

The online version contains supplementary material available at 10.1186/s13293-024-00602-6.

## Highlights

- First sex- and statin-stratified GWAS of PCSK9 plasma levels comparing SNP effects in eight subgroups.
- 11 associated loci (p < 1 × 10^–6^), including six loci known for association with PCSK9 or lipids, and five novel loci independent of lipids.
- Five loci with significant sex-interactions, and seven loci with statin-interactions.
- The *PCSK9* gene was associated in all subgroups, and there were both significant sex- and statin-related effects.
- The Mendelian Randomization using four independent *PCSK9* variants resulted in significant causal estimates for all subgroups. The causal estimates of statin-treated individuals were significantly lower than those of statin-free participants. This difference increased testing the subgroup of men, and decreased in women.

**Supplementary Information:**

The online version contains supplementary material available at 10.1186/s13293-024-00602-6.

## Background

Proprotein convertase subtilisin/kexin type 9 (PCSK9) is an protein that binds to low-density lipoprotein receptors (LDL-R) on hepatocytes, inducing their degradation [1]. As a consequence, cellular uptake of low-density lipoprotein cholesterol (LDL-C) is reduced resulting in increased serum/plasma levels [1]. This mechanism qualifies PCSK9 as a key element in lipid metabolism and therapeutic target to treat hypercholesterolemia in addition to known cholesterol-lowering drugs such as statins. PCSK9 inhibitors such as evolocumab [2], alirocumab [3], and inclisiran [4] are indicated in case the targeted LDL-C reduction by statins or other cholesterol-lowering drugs cannot be achieved. However, the effect of statin treatment is attenuated to some extent by a feedback mechanism involving PCSK9. In detail, reduced intracellular levels of cholesterol activate the transcription factor SREBP-2, which increases gene-expression of both *LDLR* and *PCSK9* [5].

Sexual dimorphisms are common in lipid metabolism, and PCSK9 also exhibits sex-differential behavior with women having higher circulating PCSK9 concentrations than men [6, 7]. While large-scale sex-stratified genome-wide association meta-analyses (GWAMA) were already performed for the common lipid traits such as: total cholesterol (TC), LDL-C, high-density lipoprotein cholesterol (HDL-C), triglycerides, and non-HDL-C [8], so far there was no GWAS on stratified PCSK9 levels to the best of our knowledge.

Sexual dimorphism are also observed for statin treatment regarding both, dose–response and likelihood to experience adverse effect [9, 10]. Although the link between statins and PCSK9 levels is well established, there is only limited data on the genetics of PCSK9 levels with vs. without statin treatment. A small GWAS (n = 562) on PCSK9 response to statin treatment detected an association at *WDR52* [11], which was not found in our earlier study analyzing statin-adjusted and statin-free PCSK9 levels [12]. In this study we identified four genome-wide significant loci, of which three were within established lipid loci, namely *PCSK9* at 1p32.3, *APOB* at 2p24.1, and *TM6SF2* at 19p13.11. Of note, *TM6SF2* was best associated in a secondary analysis of individuals without statin treatment. However, as we tested the best variant per locus for sex- and statin-interaction, we might have missed possible sex- or statin-specific loci in our previous analysis.

In the present study, we performed the first sex- and statin-stratified GWAMA on PCSK9 levels in a total of 20,016 individuals of European descent (statin-free women: n = 7183; statin-free men: n = 7642; statin-treated women: n = 1753; statin-treated men: 3438). Using this approach, we aimed at identifying both 2-way interactions of ${\text{SNP}}\times {\text{sex}}$ and ${\text{SNP}}\times {\text{statin}}$. This allowed us to characterize the well-established *PCSK9* gene locus regarding sex- and statin-specific effects, and the detection of novel loci regulating PCSK9 levels in specific subgroups. We used the identified genetic associations at the *PCSK9* gene locus to analyze strata-specific causal effects of PCSK9 on LDL-C by Mendelian Randomization analysis.

## Material and methods

### Studies and PCSK9 measurement

For this stratified GWAMA, six European-based studies contributed genome-wide summary statistics, namely *LIFE-Heart* [13], *LIFE-Adult* [14], *LURIC* [15], *TwinGene* [16], *KORA-F3* [17], and *GCKD *[18]. A brief description of studies is given in the Additional file 1 and Additional file 2: Table S1. Detailed information regarding PCSK9 measurements, genotyping and study level quality control can be found elsewhere [12, 19]. *LIFE-Heart, LIFE-Adult*, *LURIC*, and *TwinGene* imputed their genetic data on 1000 Genomes Phase 3 [20], while *KORA-F3* and *GCKD* were imputed on the Haplotype Reference Consortium panel (HRC r1.1 2016) [21]. All studies used hg19 base positions. All studies meet the ethical standards of the Declaration of Helsinki and were approved by relevant institutional review boards. Written informed consent including agreement with genetic analyses was obtained from all participants of all studies.

From each study group, we requested genome-wide association statistics for log-transformed PCSK9 levels of the four subgroups of men and women with and without statin treatment. Genetic association analysis was performed assuming additive regression models adjusting for age, active smoking, and genetic principal components (if necessary). We included chromosome X assuming total X-inactivation with male genotypes coded as A = 0 and B = 2. Associations were run with PLINK2 [22] (*LIFE-Adult, LIFE-Heart, LURIC*), REGENIE [23] (*KORA-F3*, *GCKD*), or GCTA MLMA LOCO [24, 25] (*TwinGene*), adjusting for the relationship of the dizygotic twins included in this study.

### Meta-analyses

For each of the four subgroups, we harmonized the study-specific summary statistics regarding SNP allele coding, and chromosomal position (hg19) as reported in 1000 Genomes [20] using the R package ‘EasyQC’ [26]. SNPs with missing information, MAF < 1%, imputation info score < 0.5, minor allele count < 6 or deviations of calculated allele frequency > 20% from the population reference were removed.

A flow chart of our meta-analysis approach can be found in Additional file 1: Fig. S1. First, we combined the single study results of the four analysis groups using fixed-effect models and calculated both sample-size weighted effect allele frequencies (EAF) and imputation info scores. We removed all association results with high heterogeneity across studies (I^2^ ≥ 90%), and those which are based on a single study only. In a second step, we pair-wisely combined the meta-analysis results to estimate overall effects for the sex groups and the treatment groups, respectively using fixed effect models. Only SNPs available for both pairs and without high across-study heterogeneity (I^2^ < 90%) were considered for that purpose. Of note, the statin-free subgroup was not used for discovery of novel PCSK9 associations, because this was already done in a recent analysis. We considered this group for genetic statin-interaction analysis only.

### Locus definition

We considered SNPs associated with p < 5 × 10^–8^ and p < 1 × 10^–6^ with any of the eight analysis groups as genome-wide and suggestive significant, respectively. Since our main focus is to identify interaction results, we considered the relaxed suggestive significance threshold for locus identification, while interaction testing was performed with a stringent cut-off (see below). We extracted all SNPs with at least suggestive significance for at least one of the analysis groups and ranked them by their lowest p-value over all analysis groups. For locus definition, we assigned the best associated SNP and all associated SNPs within 500 kb around its base position to the first locus. This was repeated for all unassigned SNPs until no SNPs remained. We collapsed overlapping loci, defined by distances of less than 1 MB between the lead variants. Loci containing less than three SNPs were discarded.

### Annotation

All valid SNPs reaching at least suggestive significance were annotated with nearby genes (Ensemble [27], ± 250 kb), expression quantitative trait loci in linkage disequilibrium (LD) (r^2^ ≥ 0.3) [28–32], GWAS Catalogue traits which are in LD (r^2^ ≥ 0.3) [33], and the deleteriousness score CADD as defined in [34]. Pathway enrichment was performed based on nearby genes and eQTL genes considering DOSE [35] and Reactome [36] pathways. We estimated the explained variance per SNP and subgroup using the formular of Shim et al. [37]: ${r}^{2}= {\beta }^{2}/\left({\beta }^{2}+N\times {SE}^{2}\right)$.

### Fine-mapping of the *PCSK9* gene locus

We performed fine-mapping for a deeper understanding of the genetics at the *PCSK9* gene locus. We analyzed the region ± 500 kb around the lead variant rs11591147, which was associated in all analysis subgroups with genome-wide significance. First, we performed conditional-joint analyses in each subgroup to identify additional independent variants at the locus using the COJO-slct function of the GCTA tool (v1.92.0beta3) [24, 38]. As LD reference panel, the merge of genetic data of *LIFE-Adult* and *LIFE-Heart* was used. As the subgroups cannot be expected to have identical independent variants, we determined the pairwise LD matrix of all identified independent variants across all subgroups [39], and defined clusters of SNPs in high pairwise LD (LD r^2^ > 0.7). This resulted in four clusters. For each cluster we selected the best-associated SNP as representative. Then, we estimated the respective joint statistics of the four SNPs in each of the subgroups (COJO-joint). One of these four SNPs was not available in statin-free individuals due to heterogeneity filtering (I^2^ > 90%). Hence, we used for this subgroup only the originally selected SNP (rs2495477 instead of rs693668, LD r^2^ = 0.706). We also estimated conditioned statistics using the COJO-cond function for later use in colocalization analyses.

### Interaction tests

We tested all lead SNPs per locus for interactions with sex and/or statin treatment. Two-way interactions were tested by comparing the effect sizes of the best-associated subgroup with its complementary groups regarding sex and statin treatment. For example, if the best-associated subgroup was statin-free men, then we tested against statin-free women (sex-interaction) and statin-treated men (statin-interaction):

$${t}_{sex}=\frac{{b}_{W}-{b}_{M}}{\sqrt{{SE}_{W}^{2}+{SE}_{M}^{2}}}\text{ and }{t}_{statin}=\frac{{b}_{free}-{b}_{treated}}{\sqrt{{SE}_{free}^{2}+{SE}_{treated}^{2}}}.$$

This allows identification of sex-differences respectively statin-related differences of genetic effect sizes [40]. For this analysis, we used all statistics even if one of them was originally filtered for heterogeneity. Multiple testing was accounted for by performing hierarchical false discovery rate (FDR) correction [41]. First, we applied Benjamini and Hochberg (BH) correction for the number of interaction tests per SNP. Then, we corrected for the number of tested SNPs using BH and the lowest q-value of the first step per SNP. The number of SNPs with significant interactions was then used to define the significance threshold of the first level: ${\alpha }=0.05\times k/l$, where $l$ is the number of tested independent lead SNPs and $k$ is the number of SNPs with at least one significant interaction [41]. We defined a SNP stratum-specific, if the interaction was significant and the SNP effect was suggestive significant (p < 1 × 10^–6^) in only one of the two tested subgroups. We use the term stratum-related, if the interaction was significant and the SNP had suggestively significant effects in both subgroups.

### Co-localization analyses

In colocalization analyses two local association profiles are tested for a shared causal variant [42]. Bayesian posterior probabilities (PP) of five hypotheses are calculated: H_0_: no association for both traits, H_1_ and H_2_: association for trait 1 respectively 2, only. H_3_: associations for both traits but different causal variants. H_4_: associations for both traits with at least one shared variant. We used as threshold PP(H_i_) ≥ 0.75 to support one of the hypotheses.

We performed three different colocalization analyses using our GWAMA and conditional statistics using the R package ‘coloc’: First, we analyzed each locus for colocalization between analysis groups to further support our 2-way interaction results on the basis of a genomic region rather than a single SNP interaction. In more detail, for a stratum-specific hit, we searched for conformation in form of high PP(H_1_), with trait 1 being the respective associated stratum. In case of a strata-related hit we expected high PP(H_4_) as there should be the same signal in both strata.

Next, we tested for co-localization between expression quantitative trait loci (eQTLs) and our PCSK9 association statistics to identify possible candidate genes acting via gene-expression. Here, we considered all annotated genes of the lead SNP of each locus. We used eQTL statistics in all tissues provided by GTEx v8 [43] for this analysis. At the *PCSK9* locus, we tested both the GWAMA and conditional statistics. Finally, we tested for co-localization between our PCSK9 signals and other traits to identify possible related outcomes. This included lipid traits [8], coronary artery disease (CAD) [44], bilirubin levels (GCST90019521) [45] and sleep duration (GCST007561) [46], as PCSK9 is linked to the circadian rhythm.

### Mendelian randomization using cis-effects

Finally, we aimed at determining the strata-specific causal effects of PCSK9 on LDL-C using Mendelian Randomization analyses (MR) [47]. As instruments for PCSK9 levels we used the four independent variants at the *PCSK9* gene locus (cis-pQTLs for PCSK9) as obtained from our GWAS. The LDL-C association statistics of the four *PCSK9* SNPs were obtained from the UK Biobank (UKBB, application 98032). In brief, we included all self-reported Whites and with LDL-C measurement at baseline, and excluded participants with relatives in the data set (as defined in data-field 22021) or with sex-mismatches between the genetic and database sex. Details on genotyping of the UKBB can be found elsewhere [48]. To assess lipid-lowering medication codes in UKBB, we used the coding table of Wu et al. [49] and filtered for ATC-coding “C10A”. We estimated the four SNP effects using PLINK 2.0 in the four double-stratified groups adjusting for age and the first 10 principal components. The sex-combined and statin-combined groups were obtained via meta-analysis as described for the main PCSK9 GWAS. We repeated the sex-stratified analysis using the summary-statistics of Kanoni et al. [8], in which LDL-C was adjusted for statin treatment. We did not repeat the statin-stratified analysis, as no GWAS data for LDL-C under statin treatment is available to our knowledge.

The three key MR assumptions are reasonably met:

1. The instruments need to be significantly and strongly associated with PCSK9 levels. The four SNPs were genome-widely associated in almost all subgroups. In statin-treated men and the combined statin-treated individuals, one of the four SNPs was only suggestively significant, but still represents a strong instrument (F-statistic > 10 for all SNPs). In women under statin treatment, three of the four SNPs were only nominally significant with F-statistics in between 7 and 10, probably due to the lowest sample size across all subgroups. Therefore, this subgroup was only used for sensitivity analysis focusing on consistent effect size and direction rather than significance.
2. The instruments need to be uncorrelated with possible confounders of the PCSK9–LDL-C relation. This could be a concern for sex and statin treatment, as SNPs were partly sex- and/or statin-specific. Therefore, we tested all subgroups in sensitivity analyses and applied a “leave-one-out” method to ensure that the observed effects were not caused by one SNP alone.
3. The instruments are not allowed to have a causal link to the respective outcome which is not mediated by the exposure. Using only cis-pQTLs at the *PCSK9* gene locus, this assumption is most likely satisfied, as these cis-variants will first affect PCSK9 directly and then the lipid metabolism via PCSK9 plasma levels as a downstream effect.

To estimate the causal effects we used the inverse-variance weighted (IVW) MR method [50] assuming fixed effects and no pleiotropy, which is reasonable for cis-instruments. We assessed potential pleiotropy with Cochran’s Q and visualized the heterogeneity in Forest Plots. We performed two types of sensitivity analyses: MR-Egger and “leave-one-out” method. MR-Egger corrects for horizontal pleiotropy [51]. In the “leave-one-out” method, we tested all combinations of three of the four SNPs to identify SNPs causing the heterogeneity. For all MR analyses, the R-package “MendelianRandomization” was used. Finally, we tested for differences in causal estimates using the 2-way interaction tests as described above.

## Results

### Overview of genome-wide and suggestive findings within analysis groups

We performed sex-, statin- and sex-statin-stratified GWAMAs of PCSK9 levels in up to six independent studies of European descent (sample size ranging between 247 for the subgroup of statin-treated women and 14,825 for the subgroup of statin-free individuals). After harmonizing the SNPs across studies and filtering for high quality variants, about 9.5 million SNPs remained across all analysis subgroups. We observed no signs for inflation, with maximum λ_GC_ of 1.01. SNP numbers and sample sizes per analysis group are summarized in Additional file 2: Table S2.

**Fig. 1:**
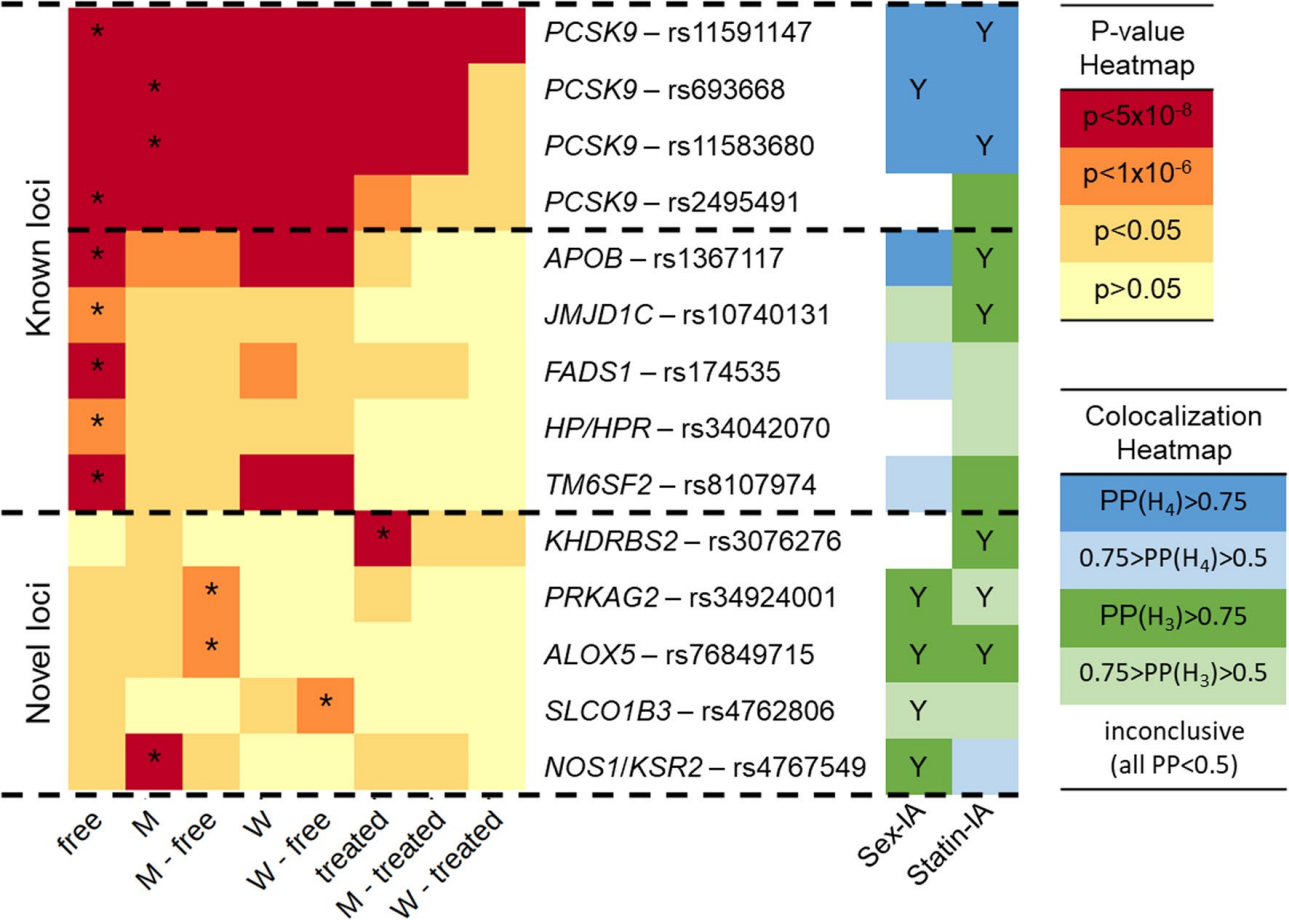
Heatmap of the log-transformed p-values of the 14 independent SNPs across all eight subgroups and of the within-PCSK9 colocalization*.* SNPs are sorted as in Table 1 (by novelty and chromosomal position). The horizontal dotted lines divide three groups of associations: the main hit at the *PCSK9* gene locus, known loci with genes involved in lipid metabolism (all best-associated in the statin-free subgroup), and novel associations detected in other subgroups. Phenotypes are sorted by sample size (free, males, women, treated), and similarity, as the statin-combined and statin-free sexes show mostly the same effects. *Subgroup with lowest p-value per SNP. Y denotes significant interaction

There were 11 loci reaching at least suggestive significance in at least one subgroup (see Table 1, Additional file 2: Table S3 for a summary of all loci, in Additional file 2: Tables S4 for annotation of significant SNPs, and in Additional file 1: Fig. S2 for a Manhattan Plot over all subgroups). This included six loci with genome-wide significant signals and five on suggestive level. Although the statin-free subgroup had the best power, two of the six genome-wide signals were found in other groups: one in males at cytoband 12q24.22 near *KSR2*/*NOS1*, and one in statin-treated individuals at 6q11.1 within *KHDRBS2* (see Fig. 1).

**Table 1:** Overview of independent SNPs reaching at least suggestive significance in our GWAMA

	Cytoband	Independent SNP	EAF	Beta	p-value	Expl Var (%)	Best subgroup	Nearest/candidate gene (kb)
Known loci	1p32.3	rs11591147	0.015	− 0.372	1.70 × 10^–144^	4.32	Free	*PCSK9* (0)
		rs693668	0.633	0.052	7.80 × 10^–39^	1.51	M	***PCSK9*** (0)
		rs11583680	0.139	− 0.051	3.98 × 10^–20^	0.76	M	***PCSK9*** (0)
		rs2495491	0.756	− 0.039	5.01 × 10^–20^	0.56	Free	***PCSK9*** (6.8)
	2p24.1	rs1367117	0.314	− 0.029	2.09 × 10^–15^	0.42	Free	***APOB*** (3.3)
	10q21.3	rs10740131	0.481	0.018	1.44 × 10^–07^	0.19	Free	***JMJD1C*** (46)
	11q12.2	rs174535	0.331	0.022	1.49 × 10^–09^	0.25	Free	***FADS1*** (16)
	16q22.2	rs34042070	0.204	0.023	8.43 × 10^–08^	0.19	Free	***HPR* (0), *HP*** (6.6)
	19p13.11	rs8107974	0.087	− 0.040	5.76 × 10^–11^	0.29	Free	*TM6SF2* (4.3)
Novel loci	6q11.1	rs3076276	0.111	− 0.079	2.31 × 10^–08^	1.22	Treated	*KHDRBS2* (0)
	7q36.1	rs34924001	0.360	0.037	4.45 × 10^–07^	0.51	M—free	*PRKAG2* (0)
	10q11.21	rs76849715	0.050	0.058	6.52 × 10^–08^	0.38	M—free	*MARCHF8* (0), *ALOX5* (140)
	12p12.2	rs4762806	0.058	− 0.077	3.44 × 10^–07^	0.56	W—free	*SLCO1B3* (0)
	12q24.22	rs4767549	0.405	− 0.022	1.41 × 10^–08^	0.29	M	*KSR2* (0), *NOS1* (60)

For each locus, we report here the genomic cytoband, beta estimate, p-value and explained variance of the best-associated SNP in the best-associated subgroup. The loci are first ordered by novelty (first 6 loci: known, last 5 loci: novel), and then by chromosomal position via cytobands. For PCSK9 only, we report the four independent variants as detected by the conditional-joint analyses. Candidate genes are defined as nearest gene or other plausible gene as identified in literature review or co-localization analyses. The distance between lead SNP and candidate gene is given in kb. Bold written genes indicate positive co-localization of the PCSK9 signal and the expression of the candidate gene in at least one tissue (PP H4 ≥ 0.75). EAF: effect allele frequency; Free: statin-free subgroup (sex-combined); Treated: statin-treated subgroup (sex-combined); M: subgroup of men (statin-combined); M-Free: subgroup of statin-free men; W-Free: subgroup of statin-free women

### Associations at the *PCSK9* gene locus

The strongest association was observed at the *PCSK9* (1p32.3) locus. All analyzed subgroups showed associations with the well-known missense mutation rs11591147 on genome-wide level. Regional association plots per subgroup are shown in Additional file 1: Fig. S3. For men, there was a secondary signal downstream of *PCSK9* (rs12758651, distance to rs11591147: 908kb). As the regions overlap, we collapse these two and used up to 7586 SNPs between base position 55005647 and 56988099 in our fine-mapping approach. Here, a total of 7 SNPs were selected as independent variants, which could be grouped into 4 clusters of high pairwise LD (r^2^ > 0.7, see Additional file 2: Table S5 for COJO results and Additional file 2: Fig. S4 for LD matrix of the seven selected SNPs). For each cluster, we selected the SNPs with the highest conditional p-values across analysis groups for subsequent analyses, namely rs11591147, rs693668, rs11583680, and rs2495491. According to this selection, for the statin-free subgroup, we detected four variants as independent contributors, and for males and women three variants each, both missing rs2495491. For statin-free men, we detected rs11591147 and rs693668, while for statin-free women, statin-treated individuals, and statin-treated men both rs11591147 and rs11583680 were detected. For statin-treated women no additional signal besides rs11591147 was detected. In the following, we summarize respective interaction results per SNP.

**Fig. 2:**
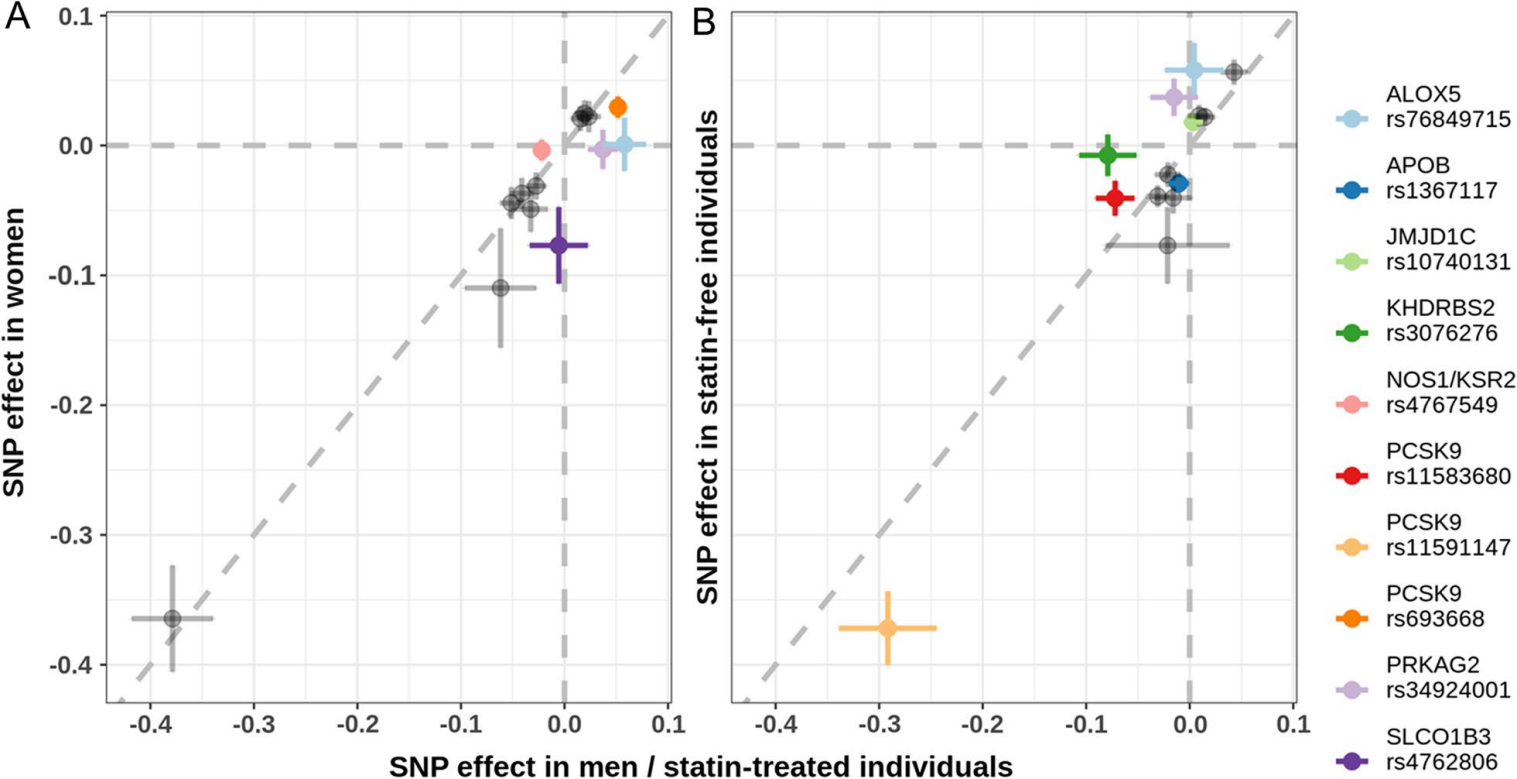
Scatter plot of beta estimates compared by interaction testing. Significant interactions are highlighted by colours and the respective candidate genes and SNPs are given in the legend on the right. Confidence intervals of SNPs without significant interaction overlap with the diagonal, stratum-specific interactions intersect with the lines x = 0 or y = 0 (grey horizontal and vertical lines), while stratum-related effects do not intersect with these lines. Panel A shows genetic sex interactions, with effect sizes in men and women at the x- and y-axis, respectively. Panel B shows genetic statin interaction, with effect sizes in statin-treated and -free individuals at the x- and y-axis, respectively

The missense mutation rs11591147 was genome-wide significantly associated in all subgroups (see Additional file 1: Fig. S5A for Forest Plot across subgroups), and the interaction test revealed a significant statin-related effect with stronger genetic effect size in statin-free individuals (q_IA_ = 8.77 × 10^–3^, see Table 2, Fig. 2 and Additional file 2: Table S6a). Colocalization confirmed that there is a unique causal variant across all strata (PP(H_4_) = 1 in all comparisons, see Additional file 2: Table S7a. Using the conditional statistics, still all group comparisons showed sufficient evidence of a shared causal signal. No significant sex interaction was observed for this variant (q_IA_ = 0.624). The genetic association signal did not colocalize with PCSK9 eQTL signals (see Additional file 2: Table S7b), but with CAD, LDL-C, TC, and non-HDL in all subgroups, and with HDL-C in all male subgroups and the two statin strata (see Additional file 2: Table S7c).

**Table 2:** Overview of ${\text{SNP}}\times {\text{sex}}$ and ${\text{SNP}}\times {\text{statin}}$ interactions significant after hierarchical FDR control

Cytoband	Candidate gene	SNP	Best subgroup	Type	$\left|\Delta \right|$	q-value
1p32.3	*PCSK9*	rs11591147	Free	Statin-related^a^	0.080	8.77 × 10^–3^
1p32.3	*PCSK9*	rs693668	M	Sex-related^b^	0.022	2.55 × 10^–4^
1p32.3	*PCSK9*	rs11583680	M	Statin-related^c^	0.031	1.56 × 10^–2^
2p24.1	*APOB*	rs1367117	Free	Free-specific	0.019	5.98 × 10^–3^
10q21.3	*JMJD1C*	rs1074013	Free	Free-specific	0.014	2.73 × 10^–2^
6q11.1	*KHDRBS2*	rs3076276	Treated	Treatment-specific	0.072	2.45 × 10^–5^
7q36.1	*PRKAG2*	rs34924001	M—free	Male-specific	0.040	1.73 × 10^–4^
7q36.1	*PRKAG2*	rs34924001	M—free	Free-specific	0.052	1.73 × 10^–4^
10q11.21	*ALOX5*	rs76849715	M—free	Male-specific	0.057	2.79 × 10^–4^
10q11.21	*ALOX5*	rs76849715	M—free	Free-specific	0.054	2.97 × 10^–3^
12p12.2	*SLCO1B3*	rs4762806	W—free	Female-specific	0.071	1.22 × 10^–3^
12q24.22	*NOS1*	rs4767549	M	Male-specific	0.018	2.46 × 10^–3^

All 14 independent SNPs were tested for 2-way interactions. We first report significant interactions at the *PCSK9* locus, then other known loci, and finally, for the novel loci identified in our meta-analysis. We only report the absolute differences of effects as the sign also depends on the choice of effect alleles. The significant interaction was denoted stratum-specific, if the SNP effect was suggestive significant (p < 1 × 10^–6^) in only one of the two tested subgroups, and stratum-related, if the SNP had suggestively significant effects in both compared subgroups. For complete results, see Additional file 2: Table S6a

^a^Stronger effect in statin-free individuals

^b^Stronger effects in men

^c^Stronger effects in statin-treated individuals

The second-strongest signal was rs693668, which was best-associated in men and also selected as independent signals in statin-free men and women (Additional file 1: Fig. S5B). For statin-free individuals, this SNP was filtered out due to high heterogeneity between the sexes (I^2^ = 91%), and rs2495477 was selected as an independent signal instead (LD r^2^: 0.706, I^2^ = 88%). We reported rs2495477 in our previous study regarding PCSK9 association in unstratified subjects (sex- and statin-adjusted) [12]. The interaction analysis revealed significant sex-interaction for rs693668, with stronger effects in men (q_IA_ = 2.55 × 10^–4^), but no statin interaction (q_IA_ = 0.099). Again, colocalization confirmed that this is a shared signal for men and women, albeit of different strength (PP(H_4_) = 0.995 comparing the statin-combined sexes). It also colocalized with *PCSK9* gene expression in liver, spleen, testis, and whole blood (see Additional file 2: Table S6a). Colocalization with LDL-C was only observed in men (PP(H_4_) = 0.896), but not in women (PP(H_4_) = 0.051).

The third-strongest independent signal was the missense mutation rs11583680, which was best-associated in men, and also conditionally significant in both statin-free and statin-treated individuals, women, statin-free women, and statin-treated men. This SNP is in weak LD to rs11206510 previously reported as independent PCSK9 association in unstratified analysis (LD r^2^ = 0.29, D’ = 0.62). We here detected another statin-interaction (q_IA_ = 0.016) with stronger effects in statin-treated men compared to statin-free men. The sex-interaction was not significant (q_IA_ = 0.399). Colocalization analysis of this signal suggest common causal variants in men and women (PP(H_4_) = 0.936), and in statin-free and -treated men (PP(H_4_) = 0.952). Colocalization analysis with other traits revealed shared signals with *PCSK9* gene expression in brain (cerebellar hemisphere and cerebellum) and pancreas tissue across subgroups. There was no colocalization with lipid traits or CAD for all subgroups having this variant as independent signal.

Finally, the fourth independent signal at the *PCSK9* gene locus was rs2495491, which was only significant in the conditional analysis of statin-free individuals, probably due to the high power to detect multiple signals (see Additional file 1: Fig. S5D). There were no significant interactions, but we detected positive co-localization with *PCSK9* gene expression for this signal for skin tissue (both sun-exposed and not sun-exposed) in men and women.

### Novel stratum-specific associations with PCSK9 levels

We detected two novel genome-wide and three novel suggestive loci in our GWAMA, all best-associated with a subgroup other than statin-free individuals which was reported in our previous work [12]. To the best of our knowledge, these new findings are not known as lipid loci so far. Here, we shortly summarize our findings regarding genetic interactions and colocalization analyses per locus. Regional Association and Forest plots are shown in Additional file 1: Figs. S6, S7.

12q24.22: The strongest novel association was detected within *KSR2* in men. There were five genome-wide and six suggestive associated SNPs at this locus, all intron-modifiers of *KSR2*. In our previous GWAS using statin-adjusted individuals, this locus only reached suggestive significance. Other traits associated at this locus comprise various sleep traits (sleep duration, daytime nap, insomnia) and height. The interaction between SNP and sex was significant (q_IA_ = 2.46 × 10^–3^) and colocalization confirmed a male-specific locus (PP(H_1_) = 0.859). We did not detect sufficient evidence for colocalization with gene-expression for the two genes nearby (*KSR2*: best PP(H_4_) = 0.495 in brain amygdala; *NOS1*: best PP(H_4_) = 0.115 in breast mammary tissue). However, there was a colocalization with a reported signal of sleep duration (PP(H_4_) = 0.984).

6q11.1: A second genome-wide signal was found within the gene *KHDRBS2*, supported by six suggestive significant SNP, which were associated within statin-treated individuals only. The lead SNP was in LD with GWAS hits for sleep traits (morning person, chronotype) and Alzheimer’s disease polygenic risk score. For the lead SNP rs3076276, we observed a significant statin-interaction (q_IA_ = 2.45 × 10^–5^). As there was no signal in statin-free individuals (PP(H_2_) = 0.810), this locus is considered specific for statin treatment. With no other gene in the vicinity, we considered *KHDRBS2* as possible candidate gene, although there were no colocalizations with eQTLs of this gene in any tissue. The signal at this locus also did not colocalize with lipid traits or sleep duration (PP(H_0_) = 0.71, as the associated SNPs were not reported in the respective sleep duration GWAS).

10q11.21: This suggestive novel locus was found near/within *MARCHF8*, *ALOX5*, and *ZFAND4*. There were 60 SNPs associated within statin-free men at suggestive level, which were also nominally associated within the subgroups of statin-free individuals and men. Several associations with blood parameters (e.g., mean corpuscular hemoglobin, red blood cell count, lymphocyte count) and carnitine levels (e.g., octadecandienylcarnitine, acylcarnitine, 3-dehydrocarnitine) were previously reported at this locus and in LD with our lead SNP. Both 2-way interaction tests were significant (q_IA_ = 2.79 × 10^–4^ for sex-interaction; q_IA_ = 2.97 × 10^–3^ for statin-interaction), which was confirmed by colocalization testing (PP(H_1_) = 0.891 comparing statin-free men and women; PP(H_1_) = 0.870 comparing men with and without statin treatment). Regarding eQTLs, we detected colocalization with *MARCHF8* gene-expression in skin tissues (PP(H_4_) = 0.829 in sun exposed and PP(H_4_) = 0.798 non exposed), and with *ZFAND4* in testis tissue (PP(H_4_) = 0.811). Considering lipid traits, we observed independent signals for TC and HDL-C here (PP(H_3_) = 0.999 for both HDL-C and TC). The respective lead SNPs of HDLC and TC are not in LD with our lead SNP (LD r^2^ = 0.025 both with rs61854123 reported for HDLC and rs2291428 reported for TC).

12p12.2: There was another suggestive locus best-associated in statin-free women around *SLCO1B3*. The 108 SNPs with suggestive significance were also associated within statin-free individuals and women at nominal level. Among the previously reported genetic associations at this locus were reduction in HbA1c levels in response to sulfonylureas treatment in type 2 diabetes and bilirubin levels. Of note, this gene was also reported for statin-induced changes in LDL-C levels [52]. The interaction test showed a significant difference of effect sizes between sexes (larger effect size in women, q_IA_ = 1.22 × 10^–3^), while the statin-interaction test was not significant (q_IA_ = 0.104). Colocalization results supported these findings, with PP(H_2_) = 0.725 comparing statin-free men and women and PP(H_1_) = 0.731 comparing women without and with statin treatment. We tested seven genes in the vicinity for eQTL colocalization but could not find any. Again, we found our signal to be independent of lipid traits (PP(H_3_) = 0.999 with logTG; PP(H_3_) = 0.815 with LDL-C) and of bilirubin levels (PP(H_3_) = 0.995), with their respective lead SNPs only in low LD with our lead variant (LD r^2^ = 0.002 with rs150266178 for LDL-C, LD r^2^ = 0.179 with rs73079476 for TG, LD r^2^ = 0.183 with rs4149056 for bilirubin).

7q36.1: Finally, there was a suggestive locus around *PRKAG2* characterized by 4 SNPs associated in statin-free men and nominal associations in all men, statin-free and statin-treated individuals. No GWAS hit was yet reported for these variants nor in LD with them. Both 2-way interactions were significant (q_IA_ = 1.73 × 10^–4^ for both). Colocalization confirmed a male-specific signal (PP(H_1_) = 0.793 comparing the statin-free men and women, PP(H_1_) = 0.743 comparing men without and with statin treatment). At this locus, we could not detect colocalizations, neither with eQTLs nor with lipid traits.

### Known associations with PCSK9 levels

Besides the associations at the *PCSK9* locus, we were able to replicate five loci previously reported for statin-free subjects or for statin-adjusted PCSK9 levels. Here we shortly add novel results regarding sex- and statin-groups and interactions. Regional Association and Forest plots are shown in Additional file 1: Figs. S8, S9.

We replicated three loci reported by Pott et al. [12] and Kheirkhah et al. [19]: 2p24.1 (*APOB*), 19p13.11 (*TM6SF2*), and 11q12.2 (*FADS1*) with genome-wide significance. At the *APOB* locus, we observed a significant statin-interaction (q_IA_ = 5.98 × 10^–3^), which was confirmed by colocalization analysis (PP(H_1_) = 0.765 comparing free and treated individuals). Although there was no significant sex-interaction, we detected colocalization with TC, non-HDL and LDL-C in women but not in men. Neither the *TM6SF2* nor the *FADS1* locus showed any significant interaction results (q_IA_ = 0.056 and q_IA_ = 0.463 for statin-interaction at *TM6SF2* and *FADS1*, respectively). For *FADS1*, we observed colocalization of our signal in statin-free individuals with sleep duration (PP(H_4_) = 0.957).

We also replicated two loci with suggestive significance previously detected by Kheirkhah et al. [19], namely 16q22.2 (*HP*/*HPR*) and 10q21.3 (*JMJD1C*, reported with suggestive significance only). While the hit at 16q22.2 did not show interactions with sex or statin, *JMJD1C* showed a significant statin-interaction (q_IA_ = 0.027), and colocalization suggested a statin-free specific signal here (PP(H_1_) = 0.768).

### Group-specific causal effects of PCSK9 on LDL-C

**Fig. 3:**
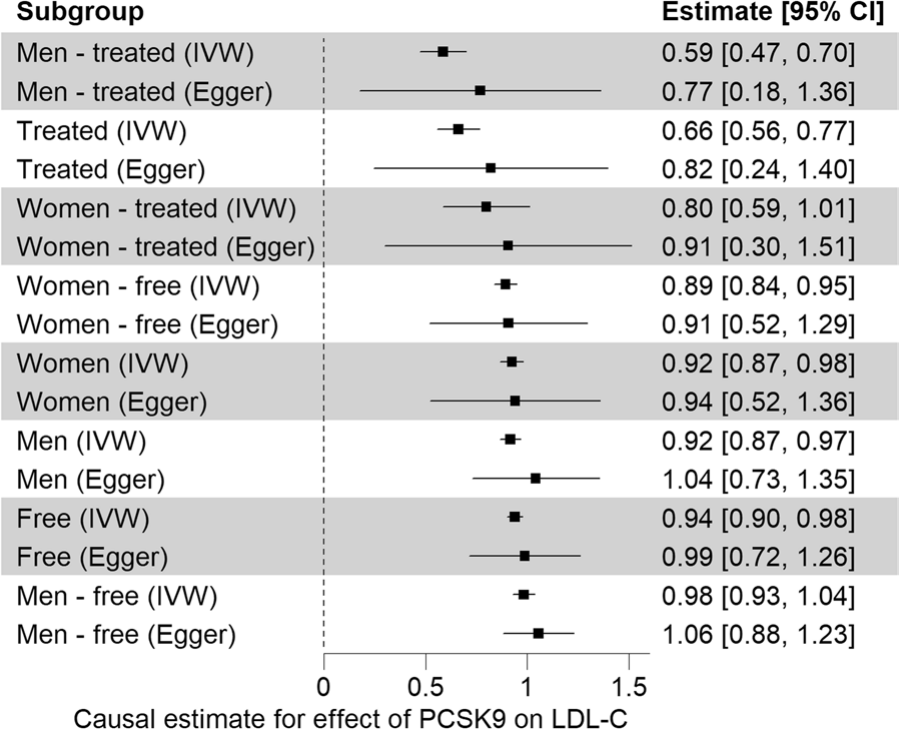
Forest Plot of the causal effects of PCSK9 on LDL-C in the eight subgroups. For each group the causal estimate and its 95% confidence interval are given. Estimates were calculated using all four independent SNPs and either the IVW method (fixed effect) or MR-Egger

Making use of the identified cis-effects of PCSK9 concentrations, we aimed at identifying stratum-specific causal effects of PCSK9 on LDL-C by Mendelian Randomization analysis. A directed acyclic graph for this causal analysis is given in Additional file 1: Fig. S10. Across all subgroups, we found significant causal estimates (see Table 3 and Fig. 3). Surprisingly, we also observed high heterogeneity of causal estimates across the four *PCSK9* SNPs in all eight subgroups (see Table 3 for Cochran’s Q and p(Q)). The same was true when using MR-Egger, although there was no significant intercept used to adjust for horizontal pleiotropy (see Additional file 2: Table S8a for F-Statistics and Wald Ratio per SNP, and Additional file 2: Table S8b for MR-IVW and MR-Egger results). A detailed analysis revealed rs11583680 to be responsible for the large heterogeneity. Despite showing the same effect direction, respective causal estimates are considerably smaller than those of the other variants (see Additional file 1: Fig. S11 for Forest plots of Wald estimates per SNP for each subgroup). Of note, this is the variant that colocalized with PCSK9 eQTLs in brain tissues, but not in liver. In the other leave-one-out models, the heterogeneity persisted on a significant level across all subgroups. Leaving out the lead SNP rs11591147 resulted in significant MR-Egger intercepts for most subgroups.

**Table 3:** Results of stratified Mendelian randomization analysis

Subgroup		All SNPs					Without rs11583680		
	min (F)	$\widehat{\theta }$	$SE\left(\widehat{\theta }\right)$	$p\left(\widehat{\theta }\right)$	Q	p(Q)	$\widehat{\theta }$	Q	p(Q)
Free	77.4	0.94	0.02	< 1 × 10^–200^	60.3	5.07 × 10^–13^	0.98	0.3	0.842
M	71.5	0.92	0.03	< 1 × 10^–200^	58.0	1.56 × 10^–12^	0.98	6.2	0.045
W	41.6	0.92	0.03	< 1 × 10^–200^	66.7	2.15 × 10^–14^	0.99	8.7	0.013
W—free	36.4	0.89	0.03	< 1 × 10^–200^	57.7	1.8 × 10^–12^	0.96	7.3	0.026
M—free	34.7	0.98	0.03	< 1 × 10^–200^	17.4	5.95 × 10^–4^	1.01	2.2	0.331
Treated	29.2	0.66	0.05	1.53 × 10^–35^	34.8	1.35 × 10^–7^	0.81	2.1	0.351
M—treated	23.7	0.59	0.06	8.00 × 10^–24^	26.7	6.78 × 10^–6^	0.74	5.0	0.082
W—treated	7.1	0.80	0.11	1.66 × 10^–13^	10.9	1.21 × 10^–2^	0.91	1.5	0.472

For﻿ each subgroup, we report the minimal F-statistics across the four instruments, and the IVW estimates using either all four SNPs (columns 3–6) or leaving rs11583680 out (columns 7–10). Cochran’s Q estimates the heterogeneity of causal estimates across instruments. After removing rs11583680, heterogeneity was markedly reduced. Full results of the other leave-one-out models and for MR-Egger can be found in Additional file 2: Table S8b

**Fig. 4:**
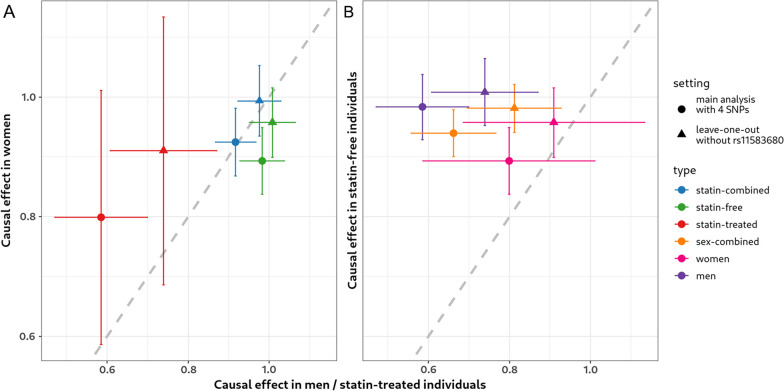
Interaction plot of MR estimates of the causal effect of PCSK9 on LDL-C. Colour indicates strata-settings, and symbols the analysis modes (circle: all four instrumental variables, triangle: rs11583680 left out). **A** Sex interactions: Only in the statin-free setting there is a difference in effect sizes between sexes (green circle). **B** Significant statin interactions: Throughout all settings, statin-free individuals had higher causal estimates than statin-treated subjects, although the difference is not significant in women (pink circle and triangle)

Next, we compared the causal effects between strata based on the results of the fixed-IVW model using all four instruments. Causal estimates were always higher in statin-free groups compared to the respective statin-treated group. The difference is significant for the sex-combined group and men only (see Table 4 and Fig. 4). In women, the difference was not significant, probably due to the small sample size we had for statin-treated women (N = 1753). However, the same trend of stronger effects in statin-free subjects was observed. Regarding sex, we found no interaction in the combined group of statin-free and statin-treated subjects, but significantly stronger effects in statin-free men than statin-free women. In the statin-treated subgroups we detected a stronger effect in women, albeit not significant. Using sex-stratified and statin-adjusted data from GLGC we repeated this MR-analysis and detected a significant interaction with stronger effects in women (statin-combined groups, ${\widehat{\theta }}_{W}$=1.26, ${\widehat{\theta }}_{M}$=1.13, p = 1.07 × 10^–7^). We repeated the interaction tests in the model leaving rs11583680 out, to ensure the interactions were not driven by the observed heterogeneity. Here, the statin-interactions remained significant, while the sex-interaction was no longer significant (p_IA_ = 0.217 in statin-free subset). However, the female-related sex-interaction in the statin-combined setting using GLGC remained significant (p_IA_ = 2.10 × 10^–9^). All results are given in Additional file 2: Table S8c.

**Table 4:** Results of interaction tests comparing causal estimates

Group 1	${\widehat{\theta }}_{1}$	$SE\left({\widehat{\theta }}_{1}\right)$	Group 2	${\widehat{\theta }}_{2}$	$SE\left({\widehat{\theta }}_{2}\right)$	$\Delta$	$SE(\Delta )$	p-value
M	0.92	0.03	W	0.92	0.03	0.01	0.04	0.858
M (GLGC)	1.13	0.01	W (GLGC)	1.26	0.02	0.13	0.02	1.07 × 10^–7^
M—free	0.98	0.03	W—free	0.89	0.03	− 0.09	0.04	0.024
M—treated	0.59	0.06	W—treated	0.80	0.11	0.21	0.12	0.083
Treated	0.66	0.05	free	0.94	0.02	0.28	0.06	1.07 × 10^–6^
M—treated	0.59	0.06	M—free	0.98	0.03	0.40	0.06	7.30 × 10^–10^
W—treated	0.80	0.11	W—free	0.89	0.03	0.09	0.11	0.401

We report and compare the results of the models with four instrumental variables using data from the UKBB. For the comparison of statin-adjusted men and women, additional data from the Global Lipids Genetics Consortium (GLGC) was available with larger sample size. The difference $\Delta$ of effect sizes was always calculated as group 2–group 1

## Discussion

In this study, we aimed at identifying sex- and statin-dependent genetic effects on circulating PCSK9 levels by performing stratified genome-wide meta-analyses in 20,016 Europeans. We detected genome-wide significant hits in all four double-stratified settings, and in all single-stratified settings. While six identified loci coincide with those found in the most recent overall meta-analysis of PCSK9 levels of statin-free and statin-adjusted subjects [12, 19], we also observed two novel genome-wide loci, one male-specific at 12q24.22, and one specific for statin-treated subjects at 6q11.1. Three additional loci with suggestive significance were found. Using a combination of interaction tests and colocalization analyses, a total of seven loci showed significant genetic sex- and/or statin-interactions.

At the known *PCSK9* gene locus on 1p32.3, we detected four independent signals across all analysis strata. Strongest associations were observed for the missense mutation rs11591147 controlling PCSK9 degradation [52]. In our analysis, this variant showed a statin-related effect with stronger effect size in individuals without treatment. One possible explanation for this interaction could be that the statin-induced increase in PCSK9 levels caps the self-degradation caused by the missense mutation, hence leading to a reduced SNP effect in statin-treated individuals. A second independent signal, rs693668, had a sex-related effect (stronger in men), and is linked to *PCSK9* gene expression in whole blood, liver and spleen. This SNP has been reported for induction of sex-biased gene-expression in whole blood by Oliva et al. [53] with a corrected p-value of 0.049. However, this sex-bias of gene expression was in the other direction: the slope is stronger in women than in men (0.25 vs 0.15). In liver tissue, no significant sex-biased eQTL was reported and also the gene expression levels of *PCSK9* were not sex-biased in any tissue in that study [53]. Regarding LDL-C levels, the SNP is sex-biased as we described it for PCSK9, with stronger effects in men (p_IA_ = 5.38 × 10^–5^ using the GLGC data [8]). In summary, this suggests sex-regulated transcription and translation of PCSK9, which could contribute to the observed sexual dimorphism of PCSK9 levels with higher levels observed in women [6]. The third independent signal, rs11583680, displayed a significant 2-way statin-interaction with the strongest effect in statin-treated men. The association signal colocalized with an eQTL signal of PCSK9 in the brain (cerebellum and cerebellar hemisphere). While we did not detect a sex-interaction, this SNP has been reported as sex-biased eQTL in both mentioned brain tissues (p_IA_ = 0.001 and p_IA_ = 0.011 for cerebellum and cerebellar hemisphere, respectively) [53]. Using the statin-stratified LDL-C data of the UK Biobank, we found a stronger SNP effect in statin-free individuals, which is contrary to our observed effect in PCSK9. One possible explanation could be that this SNP regulates PCSK9 levels predominantly in the brain. This is supported by the missing colocalization between the conditional statistics for rs11583680 and lipids and would also explain the heterogeneity caused by this SNP in the MR analyses, as corresponding causal estimates were markedly reduced compared to the other variants. The statin-interaction in combination with the function of PCSK9 in the brain needs to be further investigated, where PCSK9 possibly plays a role in neuronal differentiation, cholesterol regulation, apoptosis, and inflammation [54, 55]. The fourth independent variant at this locus, rs2495491, showed no interactions.

We used the four independent variants at *PCSK9* as instruments in an MR analysis to identify sex- or statin-related causal effects of PCSK9 on LDL-C. Indeed, we found significantly stronger causal effects in statin-free individuals and statin-free men compared to their statin-treated counterparts. In women, this reduction in effect size was not significant. This could be either due to reduced response to statin treatment, lower dosages in women compared to men, or due to the lower power given the small sample size for statin-treated women. The observed reduction of causal effect sizes during statin treatment is expected since the natural regulation of PCSK9 is superimposed by statin treatment. In detail, statins increase *LDLR* gene expression, with *PCSK9* co-activation as a side effect [5]. Hence, LDLR activation is stronger than that of PCSK9, this leads to a shift in the ratios and a decreased causal impact of PCSK9 on LDL-C (see Additional file 1: Fig. S10 for DAG).

Regarding sex, the results were mixed, with stronger causal effects in statin-free men, similar effects in the statin-combined settings, and weaker effects in statin-treated men compared to women, which again might be due to the lower power in statin-treated women. Using GLGC as additional data source, we detected a stronger causal effect in statin-combined women compared to men, in both the main analysis as well as the leave-one-out sensitivity analysis. Recent real-world studies of patients receiving PCSK9 inhibitor treatment compared the reduction of LDL-C between the sexes [56–58], and found consistently stronger relative reductions in men. Interestingly, the absolute LDL-C reduction was rather similar [56]. The baseline characteristics of these patient cohorts differ from the population-based studies included in this analysis. For example, LDL-C was reported higher in female patients, while in the general population, pre-menopausal women tend to have lower LDL-C levels than men, and the difference decreases with menopause [59, 60]. The MR estimate of our study is more similar to the absolute reduction, and indeed we did not find a sex-interaction in the statin-combined setting. The comparison of the treated subgroups might be influenced by weak instrument bias of the statin-treated women. However, our result in the statin-free subgroups is pointing in the same direction as the analysis of Myasoedova et al. [57], suggesting that the observed sex-interaction in the real world studies might be in part of genetic origin.

We detected five novel loci with stratum-specific effects, of which two reached genome-wide significance in at least one of our subgroups considered. At 12q24.22 we observed a male-specific signal, supporting our previously found suggestively significant signal from a meta-GWAS of statin-adjusted PCSK9 levels [12]. A possible candidate gene is *KSR2*, encoding the protein kinase suppressor of Ras 2. KSR2 is an important regulator of energy intake and mutation in this gene have been linked to obesity and insulin resistance [61]. It functions as a scaffold protein in the MAPK signaling pathway, regulating gene expression. A sex-differential effect has not yet been reported for this protein and needs to be further investigated. One possible mechanism could be via the protein CNKSR2, the connector enhancer of KSR2. The CNKSR2/KSR2 complex may act as mediator between MAPK, Pi3K and insulin pathways [62], offering a molecular link for the observed correlation between PCSK9 levels and insulin sensitivity indices [63, 64]. The *CNKSR2* gene is located at the non-pseudoautosomal region of the chromosome X and loss-of-function mutations are more frequent in men [65], which could affect the CNKSR2/KSR2 complex. However, chromosome X was not analyzed in detail in this study, as only two of six participating studies had chromosome X data available. More detailed analyses of the gonosomes might clarify the observed association and sex-bias.

To detect additional sex- and statin-interactions, we analyzed loci with suggestive significance in at least one of the subgroups considered. Error control of genetic interaction analyses accounted for these additional tests such that the global false positive rate of interaction reports is maintained. Thus, we tested three further loci for interaction. Accordingly, we identified a locus at 10q11.21 only associated in statin-free men, which is independent of other lipid associations at this region. Although not colocalized with eQTL signals, we considered *ALOX5* as a plausible candidate gene here. Arachidonate 5-lipoxygenase plays a role in the synthesis of leukotrienes, which are important mediators of inflammatory processes [66]. ALOX5 is known for its sex-biased translocation: in women it is cytosolic in leukocytes, while in men androgens induce its translocation into the nucleus [67]. In addition, studies in ApoE knockout mice showed that statin treatment inhibits the ALOX5 pathway [68]. In a Phase I trial of an ALOX5 inhibitor in male participants under statin treatment, a slightly decreased statin uptake was observed [69]. In vitro, this ALOX5 inhibitor weakly inhibited the hepatic statin uptake transporter SLCO1B1 [69]. Interestingly, the gene region of *SLCO1B1*, 12p12.2, was also associated with PCSK9 levels, but only in statin-free women. *SLCO1B1* mutations have been reported with statin-induced LDL-C change [70] due to a declined activity of the transporter, leading to poor transport of statins into hepatocytes [52]. The ODYSSEY OUTCOME study, which evaluated the efficacy and safety of the PCSK9 inhibitor alirocumab, found no association between a *SLCO1B1* missense mutation and statin-associated muscle symptoms, the most frequently reported adverse statin effect [58]. However, only 25% of their study population was female, which might have led to a missed association, highlighting the necessity of further sex-stratified and statin-free analyses to better understand the crosstalk between ALOX5, SLCO1B1 and PCSK9.

Finally, we were able to replicate five known loci, of which three were also significant in other subgroups than statin-free individuals considered in the most recent meta-GWAS of PCSK9 [19]. All five loci are also known for associations with other lipid traits. *APOB* has first been reported for PCSK9 association in a statin-adjusted model by our group [12]. But in the present study, we found a significant statin-interaction of this association. There was also a significant statin-interaction for *JMJD1C*, and a respective trend for *TM6SF2*, which however failed significance after correcting for multiple testing. This might suggest further studies of context-dependent genetic associations of lipid traits such as lipid treatment.

Of note, three loci were colocalized to sleep phenotypes such as sleep duration or morning person (*KSR2*, *FADS1*, and *KHDRBS2*), suggesting a genetic component for the diurnal rhythm of PCSK9 serum levels [71]. In addition, six loci were linked to insulin resistance, type 2 diabetes, or reduction in HbA1c levels in type 2 diabetes (*APOB*, *HPR*, *TM6SF2*, *KSR2*, *JMJD1C*, and *SLCO1B1*). This might contribute to the ongoing discussion of PCSK9 inhibitors increasing the risk of diabetes [72].

## Limitations

Our study has certain limitations. First, we only included Europeans. It is known that the missense mutation rs11591147 is not present in Africans or Asians. Hence the observed heterogeneity and causal estimates might not be valid for these ethnicities. Second, the sample size for statin-treated subgroups was rather low, reducing the power to detect genetic effects and colocalizations for them. Also, information on statin dosage was not available for the majority of the participating studies. Third, we could not validate the biological impact of the detected trans-QTLs. The detected lipid genes are plausible candidates due to their known impact on lipid metabolism, and two of the novel loci have been linked to statin-response. Hence those genes could indirectly affect PCSK9 plasma levels. However, the mechanism behind the other novel associations remains unclear. Further stratified analyses of larger sample sizes including ethnicities other than Europeans are necessary to validate the detected candidate genes and to corroborate and extend the observed genetic sex- and statin-interactions of PCSK9 plasma levels.

## Perspective and significance

Our study provides insight into the molecular mechanisms driving the differences in PCSK9 levels between the sexes, with impact on the strength of causal MR effect estimates in the subgroups. At present, doses of PCSK9 inhibitors are not specific for sex or statin use. Although the MR estimates mimic the life-long effect rather than treatment effects, we detected a similar sex-interaction in the subgroups without statin-treatment as others observed for PCSK9 inhibitors in patients under statin treatment or statin-intolerant. We also detected a significant statin-interaction that could depend on statin-dosages, which were not provided in the studies used here. Further studies in cohorts with information on statin doses are required to establish potential clinical significance. Additionally, our study showed that stratified MR could give meaningful estimates in subgroup analyses, if the same strata were applied to both exposure and outcome. This will be of relevance in MR studies to identify drug targets. With the increasing number of publicly available sex-stratified GWAS summary statistics, the stratified MR approach might allow researchers to identify potential sex-related drug target effects before or during drug development.

## Conclusion

We performed the first sex- and statin-stratified GWAS of PCSK9 levels in Europeans, and detected both strata-related associations at the *PCSK9* locus and strata-specific effects at seven other loci. We identified two novel genome-wide associations specific for men respectively statin-treated individuals. Besides the link to lipid metabolism, we discovered or supported plausible connections of genetic regulation of PCSK9 to other traits such as circadian rhythm and type 2 diabetes. Our Mendelian Randomization analysis indicated stronger causal effects in women and in statin-free individuals, highlighting the potential for sex-specific dosages of PCSK9 inhibitors. Pathomechanistic and clinical implications of the discovered interactions need to be further investigated.

### Supplementary Information

**Additional file 1**: **Table S1.** Study description. For each study, we report the most relevant parameters regarding our analysis. This includes description of PCSK9 measurement, covariable summary, genotyping information and GWAS tools and adjustment models. **Table S2.** Overview of sample sizes, SNP numbers and inflation factor per subgroup. For each subgroup, we report the number of valid SNPs after filtering for MAF, imputation info score and heterogeneity, the maximal number of studies available and the minimum and maximum sample size. The inflation factor λ_GC_ was estimated on the valid SNPs. **Table S3.** Overview of all associated loci (p < 1 x 10^-6^). Here we list all loci with at least one significant association. The default range of a region was 1MB (lead SNPs position +/- 500 kB), but in case of overlapping regions we used the combined range. Loci were considered valid if there were 3 or more SNPs associated within the region. Statistics are given for the best subgroup only. **Table S4a.** Annotation of all associated SNPs at valid loci (p<1x10^−6^ and 3 or more associated SNPs). For all SNPs, we report cytoband, SNP information (effect allele, EA; other allele, OA; EA frequency, EAF, and info score), compact gene information (more details in S4b-d), and statistics with all PCSK9 subgroups. **Table S4b.** Look-up of GWAS Catalogue entries of lead SNPs and their LD proxies. We searched the GWAS Catalogue for entries of the lead SNPs or their LD proxies (LD r^2^>0.3) and report here the corresponding SNP, pairwise LD, reported genes and GWAS publication. **Table S4c.** Look-up of eQTLs of lead SNPs and their LD proxies. We searched publicly available eQTL databases for entries of the lead SNPs or their LD proxies (LD r^2^>0.3) and report here the corresponding SNP, pairwise LD, reported genes, tissues, and eQTL publication. **Table S4d.** Look-up of proximate genes of lead SNPs. We report here all genes within 250 kB of the lead SNPs with the gene description, distance to the lead SNP, and gene position and orientation. **Table S5a.** Results of GCTA COJO select at *PCSK9 *gene locus (best SNP per subgroup). We searched for independent signals in each subgroup and report here the GWAS effects (column I-K) and the joint effects using LIFE as reference data (column M-P). **Table S5b.** Results of GCTA COJO joint at *PCSK9 *gene locus (same four independent SNPs for all subgroups). After selecting four independent SNPs out of the 7 selected SNPs, we estimated the joint effect of these four SNPs in all eight subgroups. The original GWAS effects are given in column F-H, while the joint effects are given in column M-O. **Table S6a.** Results of interaction test of all independent SNPs. Each independent SNP was tested for sex- and statin-interaction. We compared with respect to the best-associated phenotype. In more detail, if the best subgroup was statin-free males, then the sex-interaction was between statin-free males and females, and the statin-interaction was between statin-free and -treated males. Respective subgroups are given in columns O and U. **Table S6b.** Results of interaction test of all associated SNPs at the *PCSK9 *gene locus. Each associated SNP at *PCSK9 *was tested for sex- and statin-interaction. We compared with respect to the best-associated phenotype. In more detail, if the best subgroup was statin-free males, then the sex-interaction was between statin-free males and females, and the statin-interaction was between statin-free and -treated males. Respective subgroups are given in columns O and U. **Table S7a. **Results of colocalization analysis between the PCSK9 subgroups. We compared the association signals between the eight subgroups. For PCSK9, we tested all combinations, as all eight subgroups were associated. For the other loci, we only tested the best subgroup and their complements as in the interaction test. The two groups to compare are given in columns B and E. The five hypothesis are as follows: H_0_: no signal in either group. H_1_: only a signal in group 1. H_2_: only a signal in group 2. H_3_: signal in both groups but no colocalization. H_4_: signal in both groups and colocalization. **Table S7b. **Results of colocalization analysis between PCSK9 subgroups and gene expression. We compared the association signals of the eight subgroups with eQTL associations obtain from GTEx v8. For PCSK9, we tested also the conditional statistics. The two groups to compare are given in columns B and C. The five hypothesis are as follows: H_0_: no signal in either group. H_1_: only a signal in group 1. H_2_: only a signal in group 2. H_3_: signal in both groups but no colocalization. H_4_: signal in both groups and colocalization. **Table S7c. **Results of colocalization analysis between PCSK9 subgroups and other GWAS traits. We compared the association signals of the eight subgroups with other GWAS studies (lipids from GLGC, CAD, and sleep duration. For PCSK9, we tested also the conditional statistics. The two groups to compare are given in columns B and D. The five hypothesis are as follows: H_0_: no signal in either group. H_1_: only a signal in group 1. H_2_: only a signal in group 2. H_3_: signal in both groups but no colocalization. H_4_: signal in both groups and colocalization. **Table 8a. **Instruments used in the Mendelian Randomization (MR) analysis. In the MR, we used the same four SNPs in all eight subgroups. Here we report first the PCSK9 statistics from our GWAS including the F-statistic for each SNP, which is an indicator for instrument strength. LDL-C statistics from the stratified UKBB analysis and the Wald ratio for each SNP are added. **Table 8b. **Results of Mendelian Randomization analysis. For each subgroup, we estimated the inverse-variance weighted causal meta-effect of PCSK9 on LDL-C, and the MR-egger estimates for causal effect and intercept. For both methods, the heterogeneity is given by Cochran’s Q. Besides the method using all four instruments we list here the “leave-one-out” models using only 3 of 4 instruments. **Table 8c. **Results of interaction test of causal estimates. We used the IVW estimates of **Table S8b** to compare the causal estimates between the sexes and statin-treatment. Columns G and K indicate the compared groups.

**Additional file 2: Figure S1. **Flowchart of the stratified genome-wide analyses. We included the data of six studies of European descent. All participating studies provided GWAS summary statistics stratified for both statin-treatment and sex. In the first round of meta-analyses, we combined the study-wise data for the double-stratified subgroups. In the second round of meta-analyses, we combined pairwise strata to estimate the single-strata SNP effects, for example statin-free and statin-treated females combined to estimate SNP effects in females. Associated loci (p<1 x 10^-6^) were then tested for sex- and statin-interactions. All loci were annotated with candidate genes and tested for colocalization with gene expression and lipid data. For the *PCSK9 *gene locus, we performed fine-mapping using GCTA COJO. Finally, we compared the causal effects of PCSK9 on LDL-C using the subgroup-specific effect estimates of four SNPs at the *PCSK9 *gene locus. **Figure S2. **Manhattan Plot of all eight subgroups (min. p-value per SNP). The y-axis was limited to 20, and all SNPs with higher values set to 20 (max. original log_10_(p) = 143.8). Color indicates the subgroup with the lowest p-value for each SNP with log_10_(p) > 6. The 11 loci with sufficient support (3 or more associated SNPs) are labeled. The red dashed horizontal line indicates genome-wide significance (p<5 x 10^−8^), while the blue dotted line indicates suggestive significance (p<1x10^−6^). **Figure S3. **Regional association plots at PCSK9 gene. For each subgroup, an RA plot is given. In all plots, the lead SNP rs11591147 is plotted in blue. SNPs in LD with this variant are plotted in yellow (LD r^2^ ranging between 0.1 and 0.5). Independent variants as identified by GCTA COJO select are encircled in red. **Figure S4. **LD-Matrix plot generated by LDlink. We included all seven SNPs that were selected as independent signals in one of the subgroups to test their pairwise LD using the European reference set. The lower triangle in red indicates LD r^2^, while the upper triangle in blue indicates D‘. There are four LD-clusters visible, and their best-associated SNPs per cluster are rs2495491, rs11591147, rs11583680, and rs693668. LD between the clusters is low (r^2^ < 0.05), and LD within the cluster is high (r^2^ > 0.7). **Figure S5. **Forest Plots of the four independent SNPs over the eight subgroups. Each SNP and subgroup are plotted using the GWAS (unconditional) beta estimates and 95% confidence intervals (CI). Subgroups are sorted by increasing beta estimates per SNP (different sorting per SNP). A) rs11591147 (lead SNP) with significant statin-interaction B) rs693668 with sex-interaction C) rs11583680 with statin-interaction (males treated vs males free) D) rs2495491 without interaction. **Figure S6. **Regional Association Plot for novel loci. We detected five novel loci: *NOS1/KSR2 *(12q24.22) in males, *KHDRBS2 *(6q11.1) in statin-treated subjects, *ALOX5 *(10q11.21) in statin-free males, *SLCO1B1 *(12p12.2) in statin-free females, and *PRKAG2 *(7q36.1) in statin-free males. **Figure S7. **Forest Plots for the novel loci over the eight subgroups. Each SNP and subgroup are plotted using the GWAS beta estimates and 95% confidence intervals (CI). Subgroups are sorted by increasing beta estimates per SNP (different sorting per SNP). A) rs4767549 (*NOS1/KSR2*) with significant sex-interaction B) rs3076276 (*KHDRBS2*) with significant statin-interaction C) rs76849715 (*ALOX5*) with significant sex- and statin-interaction D) rs4763806 (*SLCO1B1*) with significant sex-interaction E) rs34924001 (*PRKAG2*) with significant sex- and statin-interaction. **Figure S8. **Regional Association Plot for known loci. We replicated five known PCSK9 loci in the statin-free subgroup. These are *APOB *(2p24.1), *TM6SF2 *(19p13.11), *FADS1/2 *(11q12.2), *HP/HPR *(16q22.2), and *JMJD1C *(10q21.3). **Figure S9.** Forest Plots for the known loci over the eight subgroups. Each SNP and subgroup are plotted using the GWAS beta estimates and 95% confidence intervals (CI). Subgroups are sorted by increasing beta estimates per SNP (different sorting per SNP). A) rs1367117 (*APOB*) with significant statin-interaction B) rs8107974 (*TM6SF2*) C) rs174535 (*FADS1/2*) D) rs34042070 (*HP/HPR*) E) rs10740131 (*JMJD1C*) with statin-interaction. **Figure S10. **Directed acyclic graph (DAG) for Mendelian Randomization (MR). We analyzed the causal effect of PCSK9 on LDL-C, stratified by sex and statin treatment. Statin treatment induces indirectly gene expression of *LDLR *and *PCSK9*. PCSK9 increases the degradation of LDLR and hence increases LDL-C plasma levels. Biological sex is a known risk factor for both PCSK9 and LDL-C. **Figure S11. **Forest Plot of the causal estimates per subgroup. In each subgroup and SNP, we estimated the Wald ration and used the first term of the delta method for the standard error. Then we combined the single SNP estimates in an inverse-variance-weighted (IVW) meta-analysis (fixed effect), and tested for heterogeneity leaving one SNP out (“w/o SNP x”). Throughout all subgroups, the causal estimate of rs11583680 is weaker than the other three introducing heterogeneity in the IVW analysis. A) Statin-free subjects B) Statin-treated subjects C) Males D) Females E) Statin-free males F) Statin-free females G) Statin-treated males H) Statin-treated females.

## Data Availability

The GWAS summary statistics for all eight subgroups generated and analyzed during the current study are available on zenodo [10.5281/zenodo.10600167]. Analysis code used during the current study is available on zenodo [10.5281/zenodo.10552659] and on github [https://github.com/GenStatLeipzig/GWAMA_PCSK9_strat]. Individual level data of the participating studies cannot be shared due to consent restrictions.
