# Supplementary material for: Sex and statin-related genetic associations at the PCSK9 gene locus: results of genome-wide association meta-analysis
Source: Biol Sex Differ. 2024 Mar 26;15:26. doi: 10.1186/s13293-024-00602-6 (PMC10964567; doi:10.1186/s13293-024-00602-6)
Supplement: Supplementary file 1 — Additional file 1: Table S1. Study description. For each study, we report the most relevant parameters regarding our analysis. This includes description of PCSK9 measurement, covariable summary, genotyping information and GWAS tools and adjustment models. Table S2. Overview of sample sizes, SNP numbers and inflation factor per subgroup. For each subgroup, we report the number of valid SNPs after filtering for MAF, imputation info score and heterogeneity, the maximal number of studies available and the minimum and maximum sample size. The inflation factor λGC was estimated on the valid SNPs. Table S3. Overview of all associated loci (p < 1 x 10^-6^). Here we list all loci with at least one significant association. The default range of a region was 1MB (lead SNPs position +/- 500 kB), but in case of overlapping regions we used the combined range. Loci were considered valid if there were 3 or more SNPs associated within the region. Statistics are given for the best subgroup only. Table S4a. Annotation of all associated SNPs at valid loci (p<1x10^−6^ and 3 or more associated SNPs). For all SNPs, we report cytoband, SNP information (effect allele, EA; other allele, OA; EA frequency, EAF, and info score), compact gene information (more details in S4b-d), and statistics with all PCSK9 subgroups. Table S4b. Look-up of GWAS Catalogue entries of lead SNPs and their LD proxies. We searched the GWAS Catalogue for entries of the lead SNPs or their LD proxies (LD r2>0.3) and report here the corresponding SNP, pairwise LD, reported genes and GWAS publication. Table S4c. Look-up of eQTLs of lead SNPs and their LD proxies. We searched publicly available eQTL databases for entries of the lead SNPs or their LD proxies (LD r2>0.3) and report here the corresponding SNP, pairwise LD, reported genes, tissues, and eQTL publication. Table S4d. Look-up of proximate genes of lead SNPs. We report here all genes within 250 kB of the lead SNPs with the gene description, distance to the lead SNP, and [file 13293_2024_602_MOESM1_ESM.pdf]

Sex and statin-related genetic associations at the *PCSK9* gene locus – results of genome-wide association meta-analysis

Stratified GWAMA of PCSK9

Pott et al.

Supplemental Data

LIFE-Adult

LIFE-Adult is a population-based cohort of 10,000 adult inhabitants of the city of Leipzig, Germany. Participants were characterized regarding life-style and environmental risk factors and clinical and subclinical signs of diseases such as atherosclerotic cardiovascular diseases, type 2 diabetes or cognition. Detailed description of the cohort can be found elsewhere (1). LIFE-Adult meets the ethical standards of the Declaration of Helsinki. The study is approved by the Ethics Committee of the Medical Faculty of the University Leipzig, Germany (Reg. No 263-2009-14122009). Written informed consent including agreement with genetic analyses was obtained from all participants.
Genotyping was performed using the Affymetrix Axiom CEU1 SNP-array technology. Variants with a call rate less than 0.97, Hardy-Weinberg Equilibrium $P < 1x10^{-6}$, and MAF < 0.01 were removed before imputation. Imputation was performed using the 1000 Genomes Project phase 3 European reference panel with IMPUTE2.

LIFE-Heart

LIFE-Heart is a cohort of patients with suspected or confirmed stable coronary artery disease or myocardial infarction collected at the Heart Center of the University of Leipzig, Germany (2). A total of about 7,000 patients were recruited. LIFE-Heart meets the ethical standards of the Declaration of Helsinki. The study is approved by the Ethics Committee of the Medical Faculty of the University Leipzig, Germany (Reg. No 276-2005) and is registered at ClinicalTrials.gov (NCT00497887). Written informed consent including agreement with genetic analyses was obtained from all participants. Patients with myocardial infarction were excluded from the present analysis.
Genotyping was performed with either Affymetrix Axiom CEU1 or Affymetrix Axiom CADLIFE. The latter is an array containing Axiom CEU as genome-wide backbone and an additional custom content of about 62,500 SNPs from CAD loci. Both datasets were merged before imputation (intersection of SNPs). Variants with a call rate less than 0.97, Hardy-Weinberg Equilibrium $P < 1x10^{-6}$, and MAF < 0.01 were removed before imputation. Imputation was performed using the 1000 Genomes Project phase 3 European reference panel with IMPUTE2.

LURIC

The Ludwigshafen Risk and Cardiovascular Health (LURIC) study is a monocentric hospital based prospective study including 3316 individuals referred for coronary angiography recruited in the Ludwigshafen Cardiac Center, southwestern Germany from 1997 – 2000 (3). Clinical indications for angiography were chest pain or a positive non-invasive stress test suggestive of myocardial ischemia. To limit clinical heterogeneity, individuals suffering from acute illnesses other than acute coronary syndrome, chronic non-cardiac diseases and a history of malignancy within the five past years were excluded. All participants were completed a detailed questionnaire which gathered information on medical history, clinical, and lifestyle factors. Fasting blood samples were obtained by venipuncture in the early morning and stored for later analyses. Information on vital status during follow-up was obtained from local registries. Death certificates, medical records of local hospitals, and autopsy data were reviewed independently by two experienced clinicians who were blinded to patient characteristics and who classified the causes of death. Study protocols were approved by the ethics committee of the "Landesärztekammer Rheinland-Pfalz" and the study was conducted in accordance with the "Declaration of Helsinki”. Informed written consent was obtained from all participants.

Samples were genotyped on the Affymetrix 6.0 array and the Illumina 200K Metabochip array. Both datasets were merged before imputation. Variants with a call rate less than 0.98, Hardy-Weinberg Equilibrium P < 5x10-4, and MAF < 0.01 were removed. Imputation was performed using the 1000 Genomes Project phase 3 reference panel with Minimac.

TwinGene

The TwinGene project, conducted between 2004 and 2008, is a population-based Swedish study of twins born between 1911 and 1958 (4). The study participants have previously participated in a telephone interview called Screening Across the Lifespan Twin Study, conducted between 1998 and 2002. To be included in TwinGene, both twins had to be alive. The zygosity of the twins was based on self-reported childhood resemblance, or by using DNA markers (for 18% of the total sample). In total, 12,591 individuals participated by donating blood to the study, and by answering questionnaires about life style and health. The study was approved by the local ethics committee at Karolinska Institutet and all participants gave informed consent.

DNA from 9,896 individual subjects was sent to SNP&SEQ Technology Platform Uppsala, Sweden for genome wide genotyping with Illumina OmniExpress bead chip (all available dizygous twins + one twin from each available monozygotic twin pair). Genotyping results for 9,836 subjects and 731,442 SNPs passed the initial lab-based quality control. SNPs were filtered when missing more than 3% information, with minor allele frequency less than 1% or deviation from Hardy-Weinberg equilibrium ($p<=1x10^{-7}$). Subjects were filtered when missing more than 3%, showing cryptic relatedness or a deviation in heterozygosity of more than five SD from the population mean. The heterozygosity of chromosome X was used to check the sex. After QC there were 9,617 individuals and 644,556 SNPs remaining. Before phasing, we

filtered duplicates and removed the pseud-oautosomal region, and aligned the dataset to the reference panel 1000 Genomes Project phase 3 using shapeit2. Imputation was then performed with Eagle2. To adjust for the relatedness structure in TwinGene, the genetic relationship matrices (GRMs) per chromosome were estimated and combined via PLINK 2.0. The genetic association with PCSK9 was then estimated with a mixed linear model leaving one chromosome out (MLM-LOCO) implemented in GCTA (5,6). Here, the analyzed chromosome is excluded from the GRM.

GCKD

The GCKD (German Chronic Kidney Disease) study is an ongoing prospective cohort study of 5,217 individuals suffering from moderate chronic kidney disease enrolled between 2010 and 2012. Details on the study design and patient characteristics have been published earlier (7). Patients were eligible if they were 18-74 years old, of Caucasian ancestry and presented with any of the following conditions: an estimated glomerular function rate (eGFR) of 30-60 ml/min/1.73 m^2 (Kidney Disease Improving Global Outcomes [KDIGO] stage G3, A1-A3) or an eGFR >60 ml/min/1.73 m^2 in the presence of overt proteinuria defined by a urine albumin-creatinine ratio (UACR) >300 mg/g or equivalent (KDIGO stage G1–G2, A3) under regular care by nephrologists. Every participant provided written informed consent. The study was approved by the ethics committees of each participating study center. Askimed, a cloud-based web platform, was used for collection and management of data (https://www.askimed.com).

Genotype data were obtained from the GCKD study using Illumina HumanOmni2.5-8 v1.2 BeadChip (Illumina, GenomeStudio, Genotyping Module Version 1.9.4). Samples were excluded if the call rate per sample was <0.97, if there was a sex mismatch or if they failed mean heterozygosity, genetic ancestry and cryptic relatedness checks. SNPs were excluded prior to imputation if the call rate was <0.96, if positions were duplicated or if they deviated from the Hardy-Weinberg equilibrium ($p < 10^{-05}$).

The Michigan imputation Server (https://imputationserver.sph.umich.edu/index.html#) (8) was used for imputing the genotypes using the Haplotype Reference Consortium panel (HRC r1.1 2016) (9).

KORA-F3

The KORA F3 study (Cooperative Health Research in the Region of Augsburg) is a follow-up study of KORA S3, which is a population-based adult cohort study and considered as random representative sample of Southern Germany (Augsburg) (10). The inclusion criteria were age 25-74, German nationality and residence in Augsburg or surrounding counties. The KORA F3 study was conducted in 2004/2005 and included 3,184 participants (11).

A written informed consent form was obtained from every participant and the Bavarian Medical Association ethics committee approved the study.

In KORA F3 study, samples were genotyped with Illumina Omni 2.5 and Illumina Omni Express array. Samples were excluded if they had a call rate <97%. SNPs were excluded if they deviated from the Hardy-Weinberg equilibrium ($p < 10^{-06}$), had a call rate <0.98, a minor allele frequency <0.01 or if they were only available on one chip.

The Michigan imputation Server (https://imputationserver.sph.umich.edu/index.html#) (8) was used for imputing the genotypes using the Haplotype Reference Consortium panel (HRC r1.1 2016) (9).

REFERENCES

1. Engel, C., Wirkner, K., Zeynalova, S., Baber, R., Binder, H., Ceglarek, U., Enzenbach, C., Fuchs, M., Hagendorff, A. and Henger, S. *et al.* (2022) Cohort Profile: The LIFE-Adult-Study. *Int. J. Epidemiol.* First published on May 28, 2022, 10.1093/ije/dyac114.

2. Scholz, M., Henger, S., Beutner, F., Teren, A., Baber, R., Willenberg, A., Ceglarek, U., Pott, J., Burkhardt, R. and Thiery, J. (2020) Cohort profile: The Leipzig Research Center for Civilization Diseases-Heart study (LIFE-Heart). *Int. J. Epidemiol.*

3. Winkelmann, B.R., März, W., Boehm, B.O., Zotz, R., Hager, J., Hellstern, P. and Senges, J. (2001) Rationale and design of the LURIC study--a resource for functional genomics, pharmacogenomics and long-term prognosis of cardiovascular disease. *Pharmacogenomics,* **2,** S1-73.

4. Magnusson, P.K.E., Almqvist, C., Rahman, I., Ganna, A., Viktorin, A., Walum, H., Halldner, L., Lundström, S., Ullén, F. and Långström, N. *et al.* (2013) The Swedish Twin Registry: establishment of a biobank and other recent developments. *Twin Res. Hum. Genet.,* **16,** 317–329.

5. Yang, J., Zaitlen, N.A., Goddard, M.E., Visscher, P.M. and Price, A.L. (2014) Advantages and pitfalls in the application of mixed-model association methods. *Nat. Genet.,* **46,** 100–106.

6. Yang, J., Lee, S.H., Goddard, M.E. and Visscher, P.M. (2011) GCTA: a tool for genome-wide complex trait analysis. *Am. J. Hum. Genet.,* **88,** 76–82.

7. Eckardt, K.-U., Bärthlein, B., Baid-Agrawal, S., Beck, A., Busch, M., Eitner, F., Ekici, A.B., Floege, J., Gefeller, O. and Haller, H. *et al.* (2012) The German Chronic Kidney Disease (GCKD) study: design and methods. *Nephrology, dialysis, transplantation : official publication of the European Dialysis and Transplant Association - European Renal Association.* First published on August 22, 2011, 10.1093/ndt/gfr456.

8. Das, S., Forer, L., Schönherr, S., Sidore, C., Locke, A.E., Kwong, A., Vrieze, S.I., Chew, E.Y., Levy, S. and McGue, M. *et al.* (2016) Next-generation genotype imputation service and methods. *Nat. Genet.* First published on August 29, 2016, 10.1038/ng.3656.

9. McCarthy, S., Das, S., Kretzschmar, W., Delaneau, O., Wood, A.R., Teumer, A., Kang, H.M., Fuchsberger, C., Danecek, P. and Sharp, K. *et al.* (2016) A reference panel of 64,976 haplotypes for genotype imputation. *Nat. Genet.* First published on August 22, 2016, 10.1038/ng.3643.

10. Holle, R., Happich, M., Löwel, H. and Wichmann, H.E. (2005) KORA--a research platform for population based health research. *Gesundheitswesen (Bundesverband der Arzte des Offentlichen Gesundheitsdienstes (Germany)),* **67 Suppl 1,** S19-25.

11. Schnitzer, F., Forer, L., Schönherr, S., Gieger, C., Grallert, H., Kronenberg, F., Peters, A. and Lamina, C. (2022) Association between a polygenic and family risk score on the prevalence and incidence of myocardial infarction in the KORA-F3 study. *Atherosclerosis.* First published on May 21, 2022, 10.1016/j.atherosclerosis.2022.05.014.

Supplemental Tables

Table S1: Study description. For each study, we report the most relevant parameters regarding our analysis. This includes description of PCSK9 measurement, covariable summary, genotyping information and GWAS tools and adjustment models.

Table S2: Overview of sample sizes, SNP numbers and inflation factor per subgroup. For each subgroup, we report the number of valid SNPs after filtering for MAF, imputation info score and heterogeneity, the maximal number of studies available and the minimum and maximum sample size. The inflation factor λ_{GC} was estimated on the valid SNPs.

Table S3: Overview of all associated loci ($p<1x10^{-6}$). Here we list all loci with at least one significant association. The default range of a region was 1MB (lead SNPs position +/- 500 kB), but in case of overlapping regions we used the combined range. Loci were considered valid if there were 3 or more SNPs associated within the region. Statistics are given for the best subgroup only.

Table S4a: Annotation of all associated SNPs at valid loci ($p<1x10^{-6}$ and 3 or more associated SNPs). For all SNPs, we report cytoband, SNP information (effect allele, EA; other allele, OA; EA frequency, EAF, and info score), compact gene information (more details in S4b-d), and statistics with all PCSK9 subgroups.

Table S4b: Look-up of GWAS Catalogue entries of lead SNPs and their LD proxies. We searched the GWAS Catalogue for entries of the lead SNPs or their LD proxies (LD r^2>0.3) and report here the corresponding SNP, pairwise LD, reported genes and GWAS publication.

Table S4c: Look-up of eQTLs of lead SNPs and their LD proxies. We searched publicly available eQTL databases for entries of the lead SNPs or their LD proxies (LD r^2>0.3) and report here the corresponding SNP, pairwise LD, reported genes, tissues, and eQTL publication.

Table S4d: Look-up of proximate genes of lead SNPs. We report here all genes within 250 kB of the lead SNPs with the gene description, distance to the lead SNP, and gene position and orientation.

Table S5a: Results of GCTA COJO select at *PCSK9* gene locus (best SNP per subgroup). We searched for independent signals in each subgroup and report here the GWAS effects (column I-K) and the joint effects using LIFE as reference data (column M-P).

Table S5b: Results of GCTA COJO joint at *PCSK9* gene locus (same four independent SNPs for all subgroups). After selecting four independent SNPs out of the 7 selected SNPs, we estimated the joint effect of these four SNPs in all eight subgroups. The original GWAS effects are given in column F-H, while the joint effects are given in column M-O.

Table S6a: Results of interaction test of all independent SNPs. Each independent SNP was tested for sex- and statin-interaction. We compared with respect to the best-associated phenotype. In more detail, if the best subgroup was statin-free males, then the sex-interaction was between statin-free males and females, and the statin-interaction was between statin-free and -treated males. Respective subgroups are given in columns O and U.

Table S6b: Results of interaction test of all associated SNPs at the *PCSK9* gene locus. Each associated SNP at *PCSK9* was tested for sex- and statin-interaction. We compared with respect to the best-associated phenotype. In more detail, if the best subgroup was statin-free males, then the sex-interaction was between statin-free males and females, and the statin-interaction was between statin-free and -treated males. Respective subgroups are given in columns O and U.

Table S7a: Results of colocalization analysis between the PCSK9 subgroups. We compared the association signals between the eight subgroups. For PCSK9, we tested all combinations, as all eight subgroups were associated. For the other loci, we only tested the best subgroup and their complements as in the interaction test. The two groups to compare are given in columns B and E. The five hypothesis are as follows: H_0: no signal in either group. H_1: only a signal in group 1. H_2: only a signal in group 2. H_3: signal in both groups but no colocalization. H_4: signal in both groups and colocalization.

Table S7b: Results of colocalization analysis between PCSK9 subgroups and gene expression. We compared the association signals of the eight subgroups with eQTL associations obtain from GTEx v8. For PCSK9, we tested also the conditional statistics. The two groups to compare are given in columns B and C. The five hypothesis are as follows: H_0: no signal in either group. H_1: only a signal in group 1. H_2: only a signal in group 2. H_3: signal in both groups but no colocalization. H_4: signal in both groups and colocalization.

Table S7c: Results of colocalization analysis between PCSK9 subgroups and other GWAS traits. We compared the association signals of the eight subgroups with other GWAS studies (lipids from GLGC, CAD, and sleep duration. For PCSK9, we tested also the conditional statistics. The two groups to compare are given in columns B and D. The five hypothesis are as follows: H_0: no signal in either group. H_1: only a signal in group 1. H_2: only a signal in group 2. H_3: signal in both groups but no colocalization. H_4: signal in both groups and colocalization.

Table 8a: Instruments used in the Mendelian Randomization (MR) analysis. In the MR, we used the same four SNPs in all eight subgroups. Here we report first the PCSK9 statistics from our GWAS including the F-statistic for each SNP, which is an indicator for instrument strength. LDL-C statistics from the stratified UKBB analysis and the Wald ratio for each SNP are added.

Table 8b: Results of Mendelian Randomization analysis. For each subgroup, we estimated the inverse-variance weighted causal meta-effect of PCSK9 on LDL-C, and the MR-egger estimates for causal effect and intercept. For both methods, the heterogeneity is given by Cochran's Q. Besides the method using all four instruments we list here the "leave-one-out" models using only 3 of 4 instruments.

Table 8c: Results of interaction test of causal estimates. We used the IVW estimates of Table S8b to compare the causal estimates between the sexes and statin-treatment. Columns G and K indicate the compared groups.

Supplemental Figures

Figure S1: Flowchart of the stratified genome-wide analyses. We included the data of six studies of European descent. All participating studies provided GWAS summary statistics stratified for both statin-treatment and sex. In the first round of meta-analyses, we combined the study-wise data for the double-stratified subgroups. In the second round of meta-analyses, we combined pairwise strata to estimate the single-strata SNP effects, for example statin-free and statin-treated females combined to estimate SNP effects in females. Associated loci ($p<1x10^{-6}$) were then tested for sex- and statin-interactions. All loci were annotated with candidate genes and tested for colocalization with gene expression and lipid data. For the *PCSK9* gene locus, we performed fine-mapping using GCTA COJO. Finally, we compared the causal effects of PCSK9 on LDL-C using the subgroup-specific effect estimates of four SNPs at the *PCSK9* gene locus.

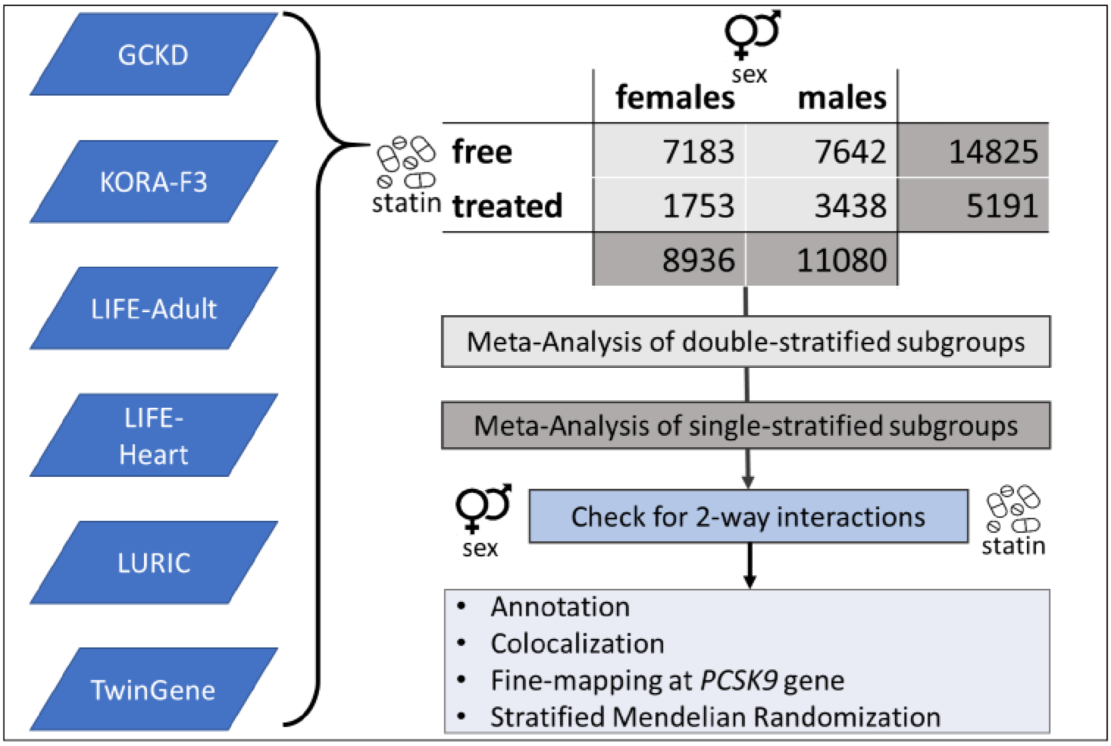

Figure S2: Manhattan Plot of all eight subgroups (min. p-value per SNP). The y-axis was limited to 20, and all SNPs with higher values set to 20 (max. original $\log_{10}(p)=143.8$). Color indicates the subgroup with the lowest p-value for each SNP with $\log_{10}(p)>6$. The 11 loci with sufficient support (3 or more associated SNPs) are labeled. The red dashed horizontal line indicates genome-wide significance ($p<5\times10^{-8}$), while the blue dotted line indicates suggestive significance ($p<1\times10^{-6}$).

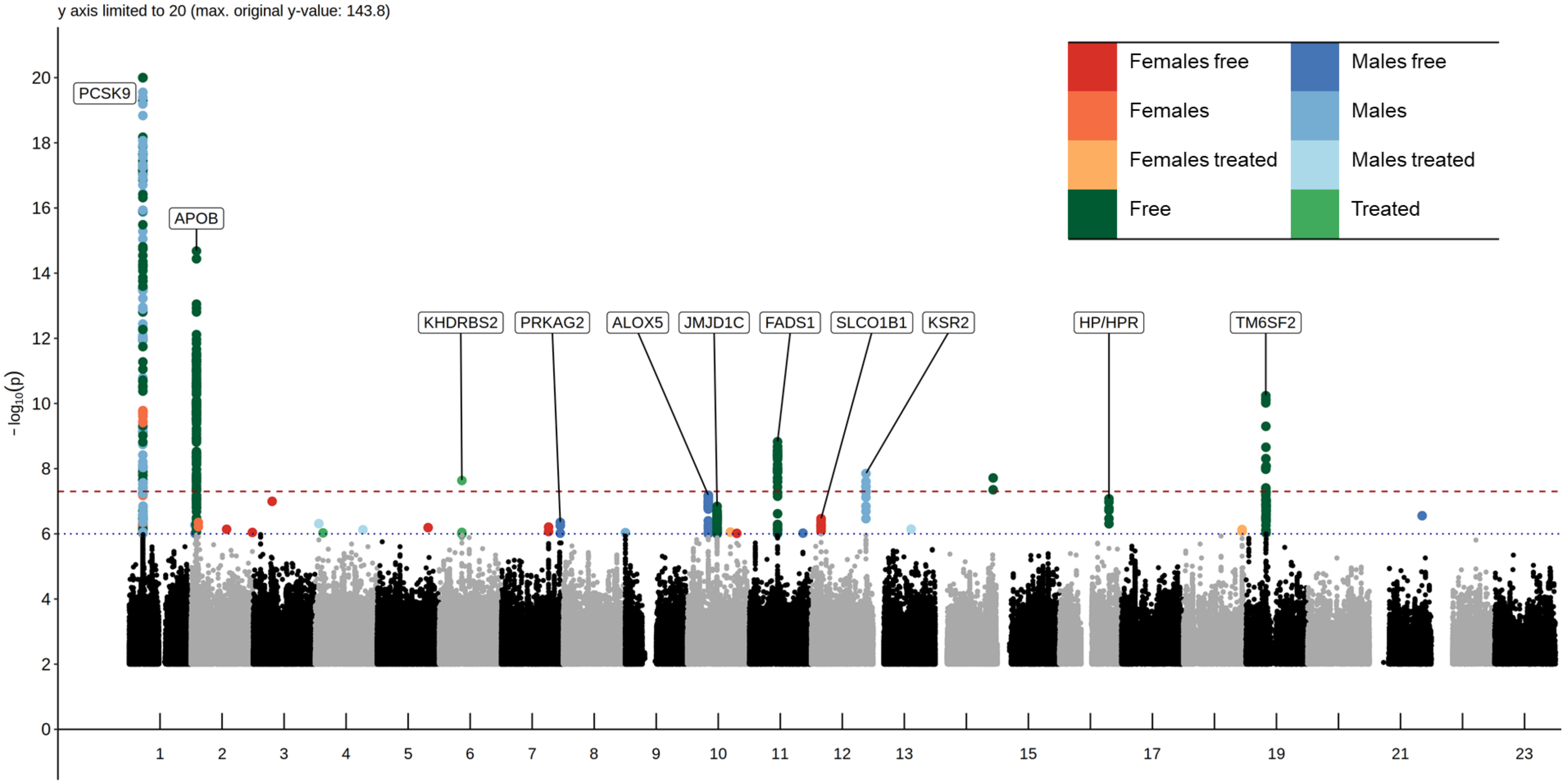

Figure S3: Regional association plots at PCSK9 gene. For each subgroup, an RA plot is given. In all plots, the lead SNP rs11591147 is plotted in blue. SNPs in LD with this variant are plotted in yellow (LD r^2 ranging between 0.1 and 0.5). Independent variants as identified by GCTA COJO select are encircled in red.

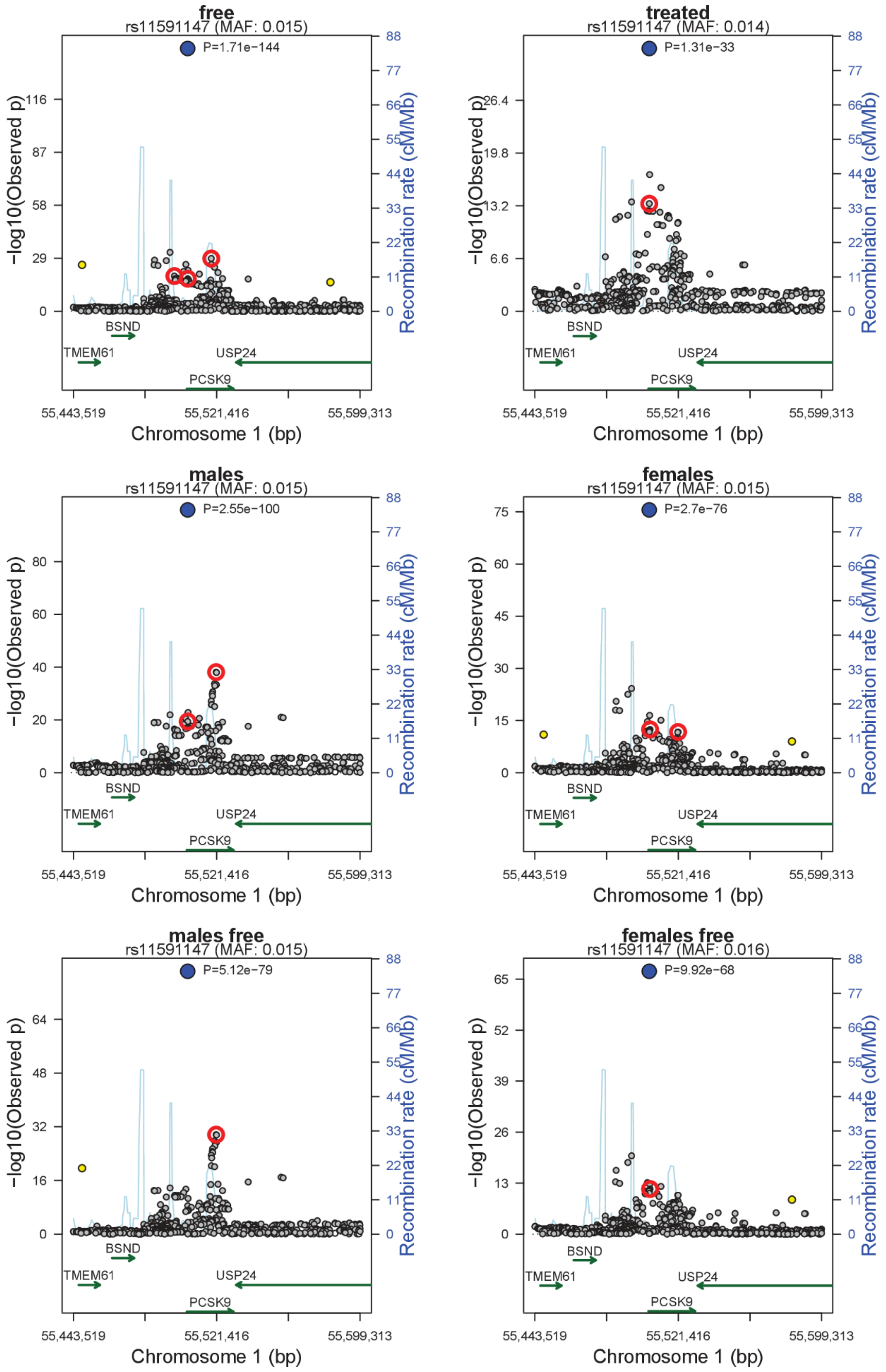

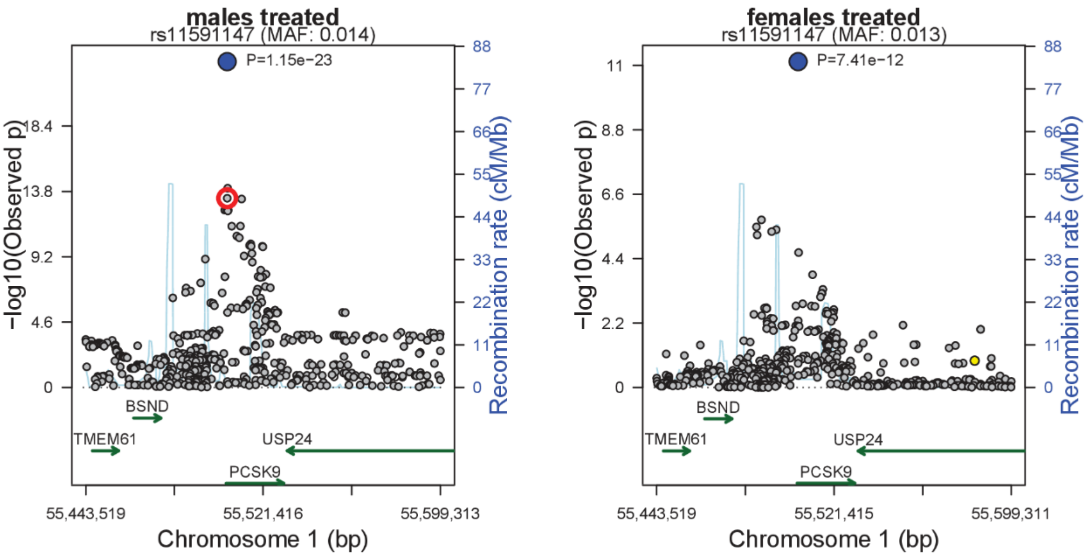
males treated
rs11591147 (MAF: 0.014)
P=1.15e−23
−log10(Observed p)
Recombination rate (cM/Mb)
BSND
TMEM61
USP24
PCSK9
55,443,519
55,521,416
55,599,313
Chromosome 1 (bp)
females treated
rs11591147 (MAF: 0.013)
P=7.41e−12
55,521,415
55,599,311

Figure S4: LD-Matrix plot generated by LDlink. We included all seven SNPs that were selected as independent signals in one of the subgroups to test their pairwise LD using the European reference set. The lower triangle in red indicates LD r^2, while the upper triangle in blue indicates D‘. There are four LD-clusters visible, and their best-associated SNPs per cluster are rs2495491, rs11591147, rs11583680, and rs693668. LD between the clusters is low (r^2<0.05), and LD within the cluster is high (r^2>0.7).

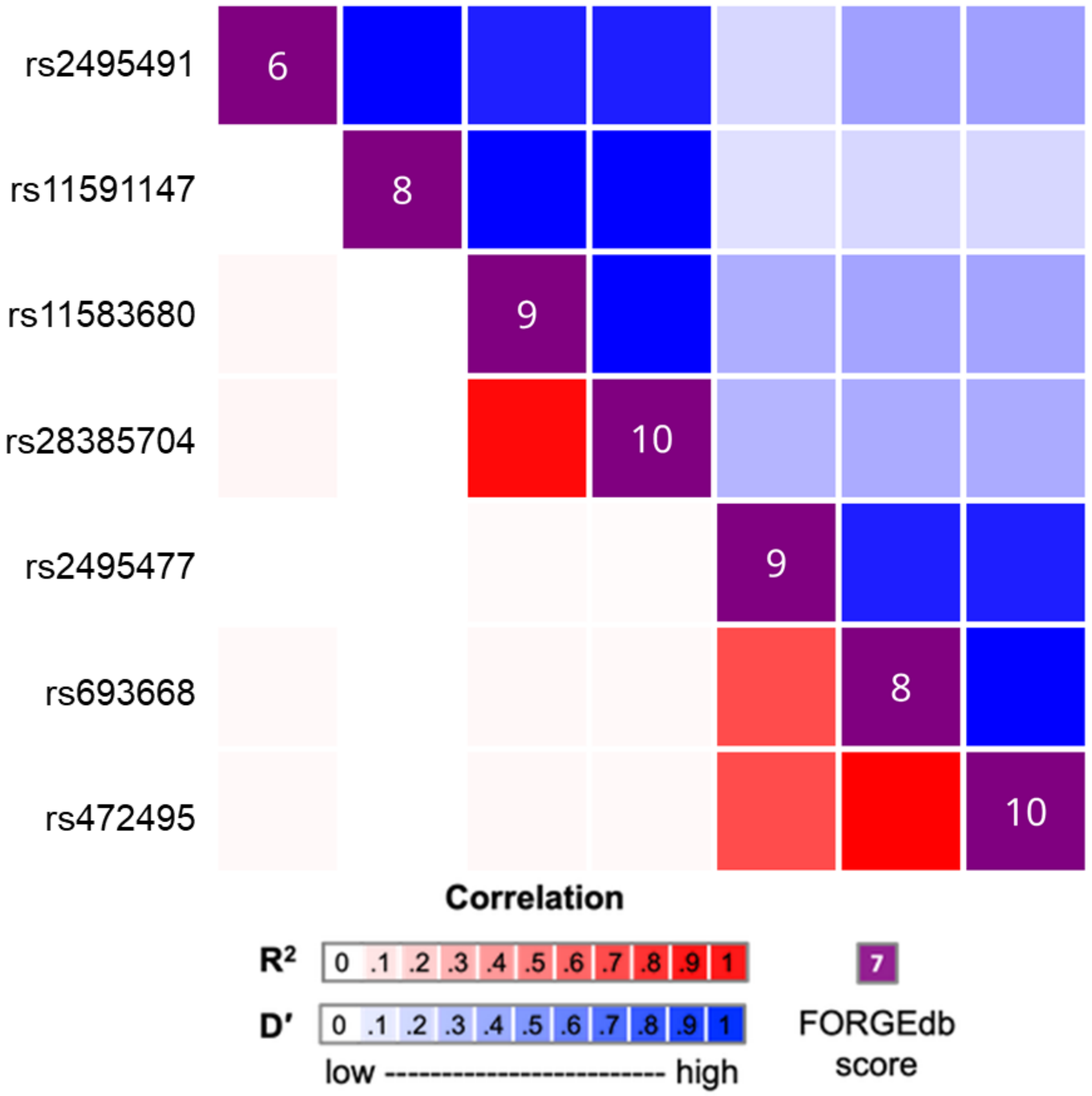

Figure S5: Forest Plots of the four independent SNPs over the eight subgroups. Each SNP and subgroup are plotted using the GWAS (unconditional) beta estimates and 95% confidence intervals (CI). Subgroups are sorted by increasing beta estimates per SNP (different sorting per SNP). A) rs11591147 (lead SNP) with significant statin-interaction B) rs693668 with sex-interaction C) rs11583680 with statin-interaction (males treated vs males free) D) rs2495491 without interaction.

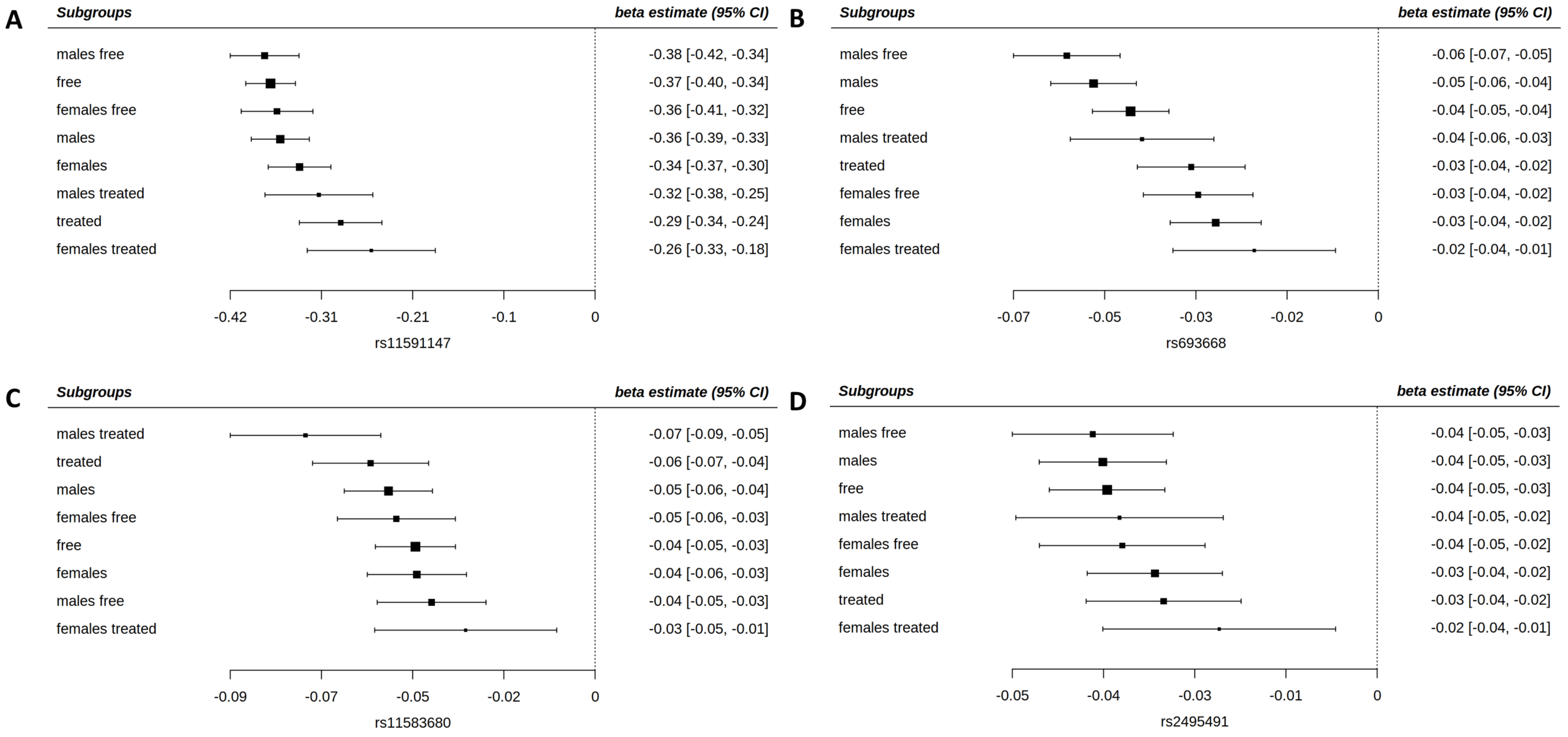

Figure S6: Regional Association Plot for novel loci. We detected five novel loci: *NOS1/KSR2* (12q24.22) in males, *KHDRBS2* (6q11.1) in statin-treated subjects, *ALOX5* (10q11.21) in statin-free males, *SLCO1B1* (12p12.2) in statin-free females, and *PRKAG2* (7q36.1) in statin-free males.

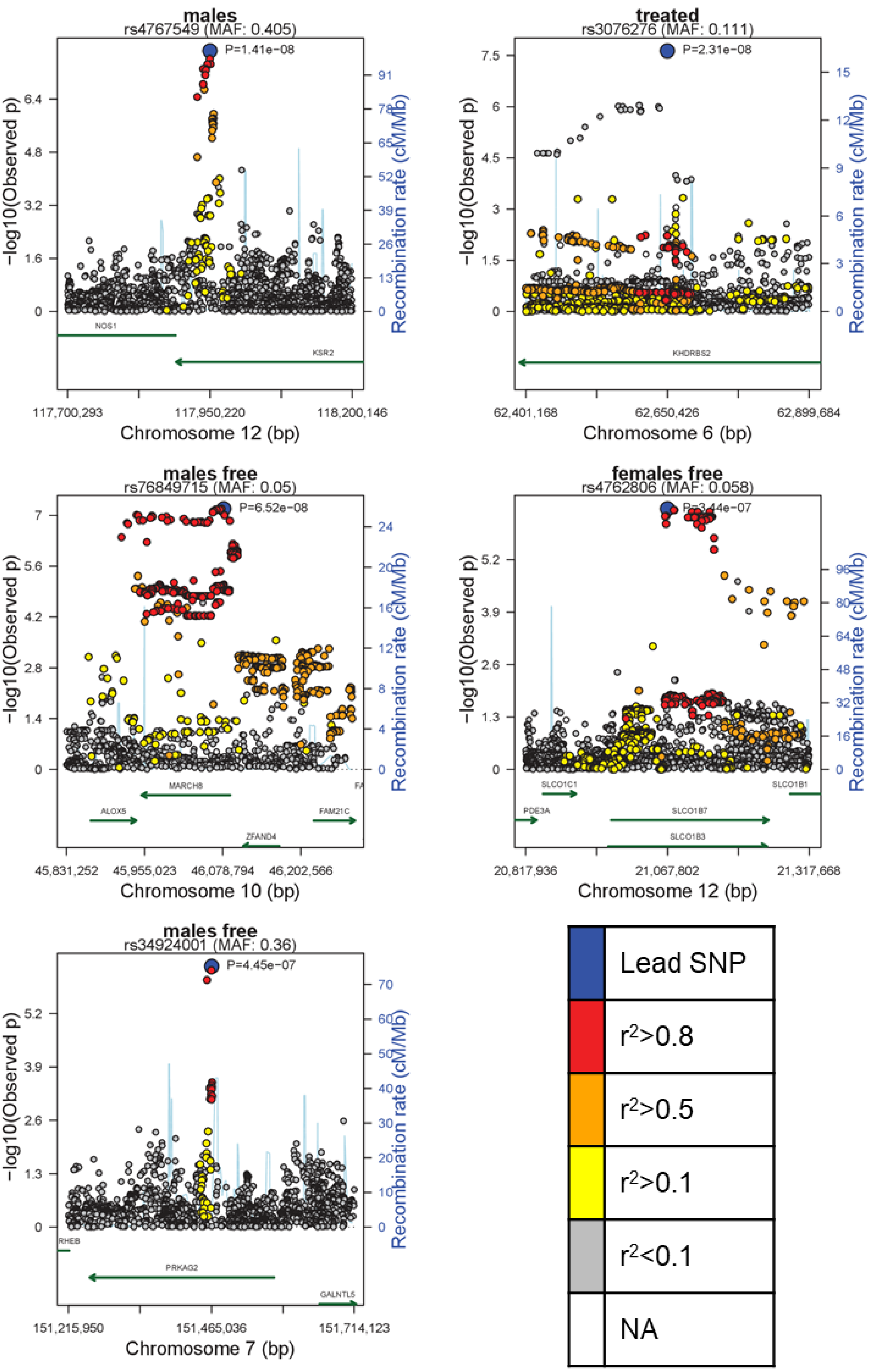

Figure S7: Forest Plots for the novel loci over the eight subgroups. Each SNP and subgroup are plotted using the GWAS beta estimates and 95% confidence intervals (CI). Subgroups are sorted by increasing beta estimates per SNP (different sorting per SNP). A) rs4767549 (*NOS1/KSR2*) with significant sex-interaction B) rs3076276 (*KHDRBS2*) with significant statin-interaction C) rs76849715 (*ALOX5*) with significant sex- and statin-interaction D) rs4763806 (*SLCO1B1*) with significant sex-interaction E) rs34924001 (*PRKAG2*) with significant sex- and statin-interaction.

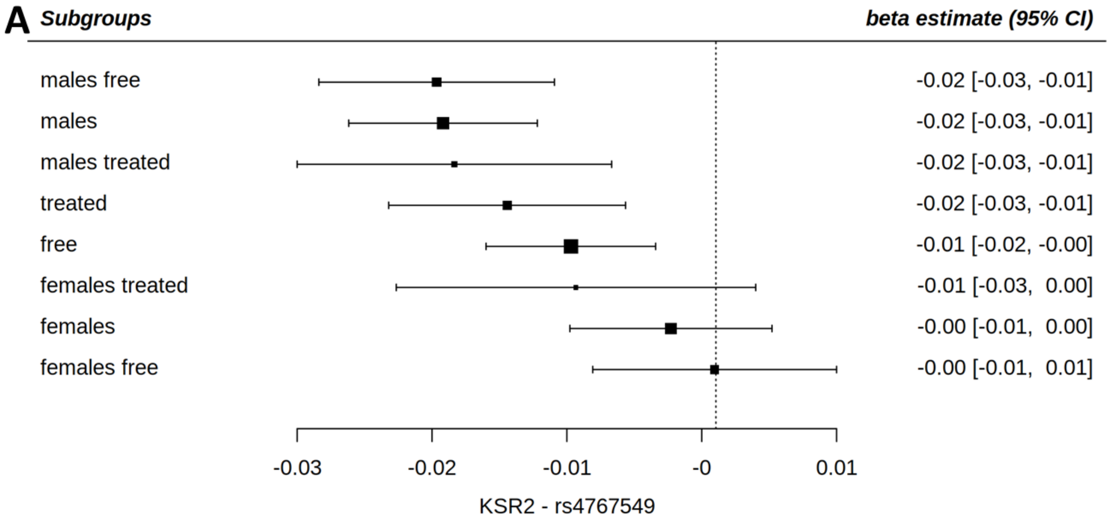

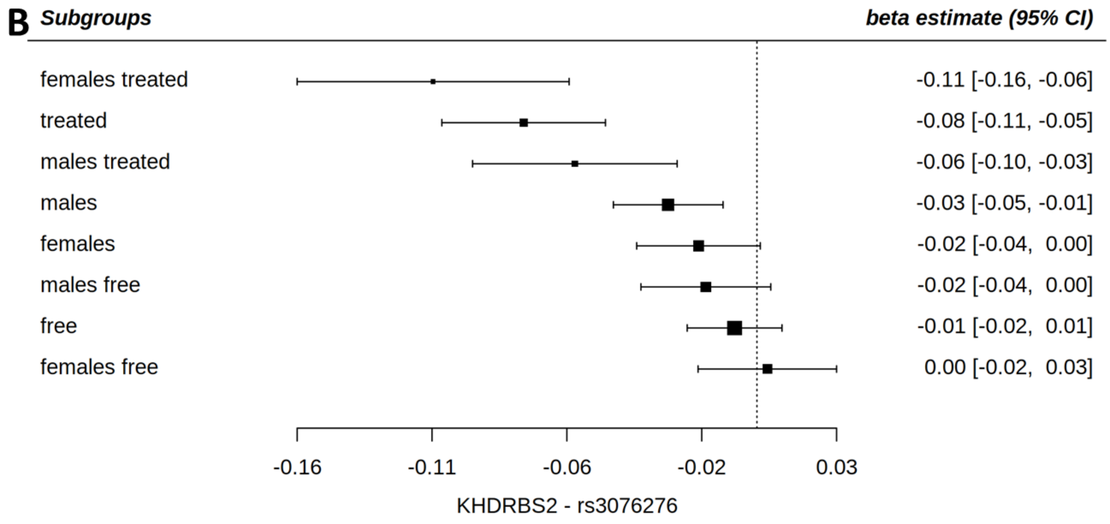

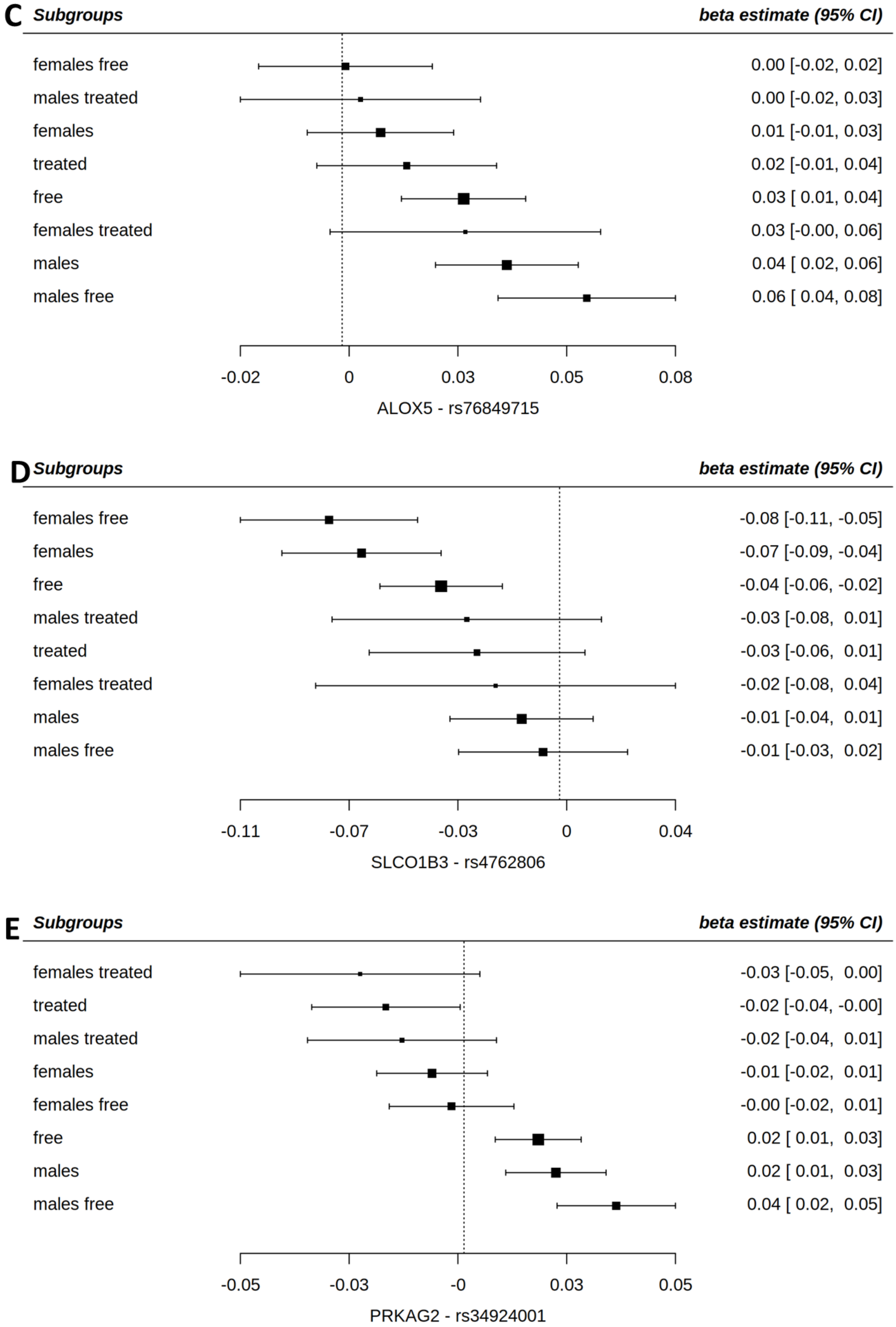
C
Subgroups
beta estimate (95% CI)
females free 0.00 [-0.02, 0.02]
males treated 0.00 [-0.02, 0.03]
females 0.01 [-0.01, 0.03]
treated 0.02 [-0.01, 0.04]
free 0.03 [0.01, 0.04]
females treated 0.03 [-0.00, 0.06]
males 0.04 [0.02, 0.06]
males free 0.06 [0.04, 0.08]
-0.02
0
0.03
0.05
0.08
ALOX5 - rs76849715
D
Subgroups
beta estimate (95% CI)
females free -0.08 [-0.11, -0.05]
females -0.07 [-0.09, -0.04]
free -0.04 [-0.06, -0.02]
males treated -0.03 [-0.08, 0.01]
treated -0.03 [-0.06, 0.01]
females treated -0.02 [-0.08, 0.04]
males -0.01 [-0.04, 0.01]
males free -0.01 [-0.03, 0.02]
-0.11
-0.07
-0.03
0
0.04
SLCO1B3 - rs4762806
E
Subgroups
beta estimate (95% CI)
females treated -0.03 [-0.05, 0.00]
treated -0.02 [-0.04, -0.00]
males treated -0.02 [-0.04, 0.01]
females -0.01 [-0.02, 0.01]
females free -0.00 [-0.02, 0.01]
free 0.02 [0.01, 0.03]
males 0.02 [0.01, 0.03]
males free 0.04 [0.02, 0.05]
-0.05
-0.03
-0
0.03
0.05
PRKAG2 - rs34924001

Figure S8: Regional Association Plot for known loci. We replicated five known PCSK9 loci in the statin-free subgroup. These are *APOB* (2p24.1), *TM6SF2* (19p13.11), *FADS1/2* (11q12.2), *HP/HPR* (16q22.2), and *JMJD1C* (10q21.3).

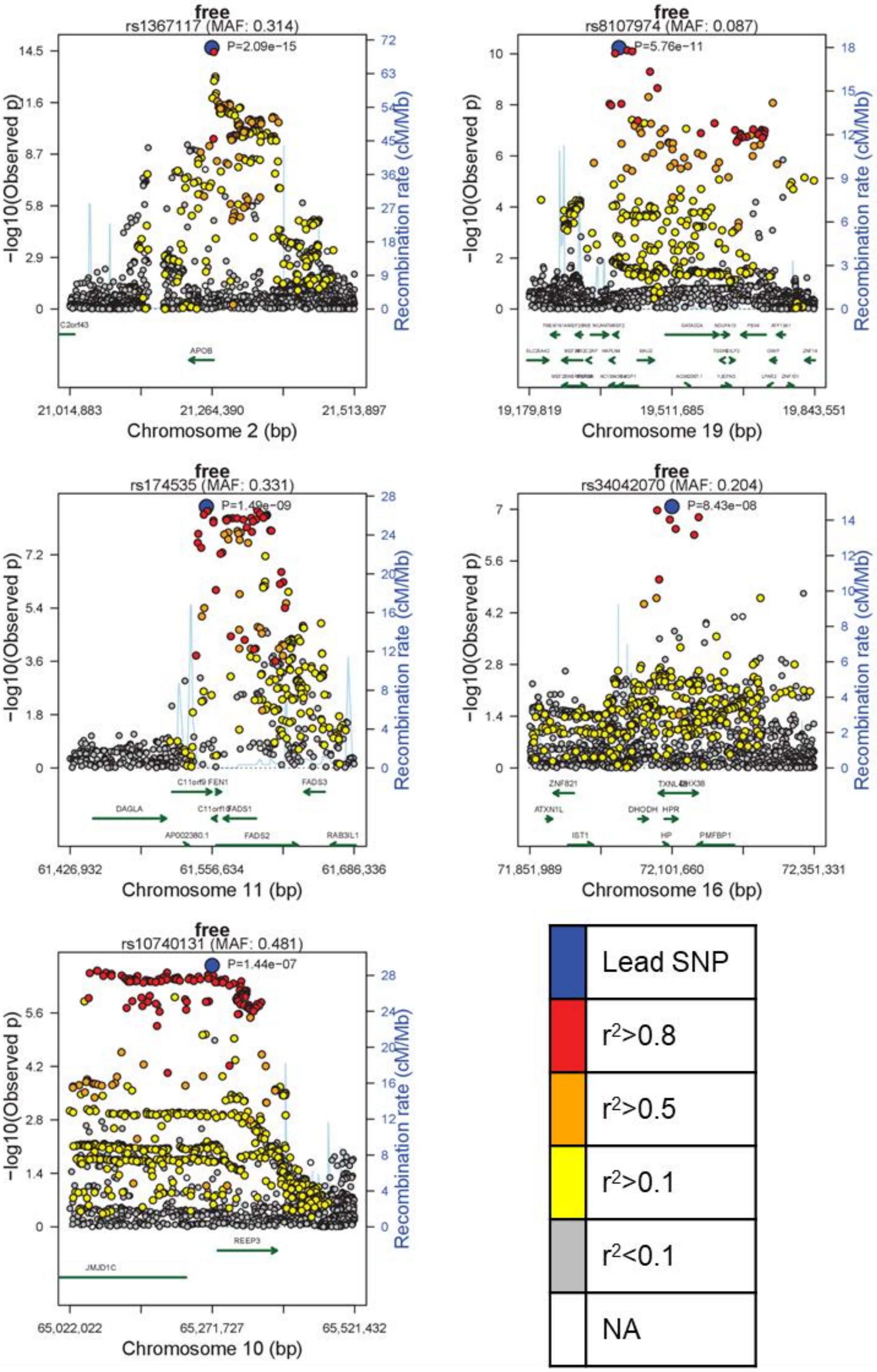

Figure S9: Forest Plots for the known loci over the eight subgroups. Each SNP and subgroup are plotted using the GWAS beta estimates and 95% confidence intervals (CI). Subgroups are sorted by increasing beta estimates per SNP (different sorting per SNP). A) rs1367117 (*APOB*) with significant statin-interaction B) rs8107974 (*TM6SF2*) C) rs174535 (*FADS1/2*) D) rs34042070 (*HP/HPR*) E) rs10740131 (*JMJD1C*) with statin-interaction.

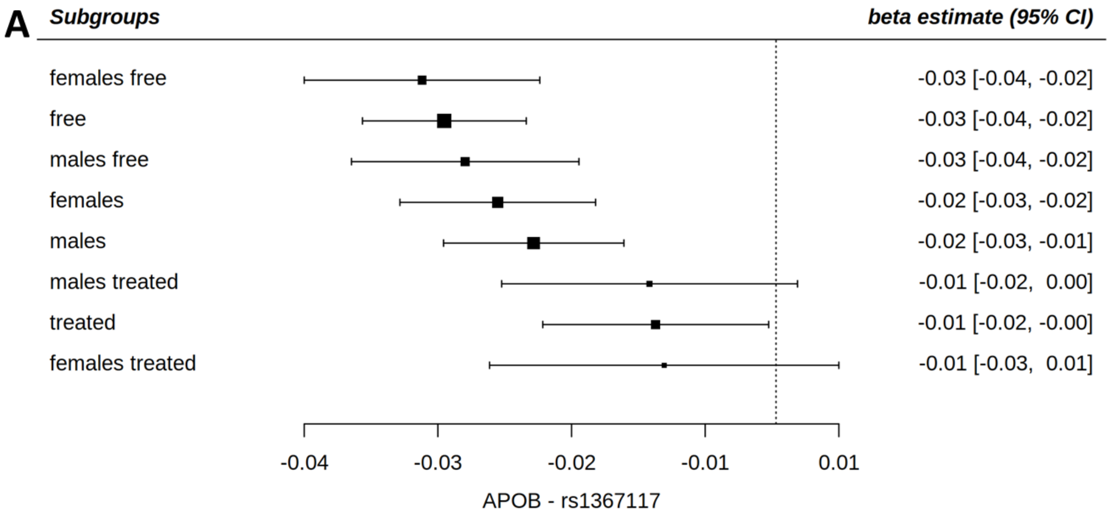

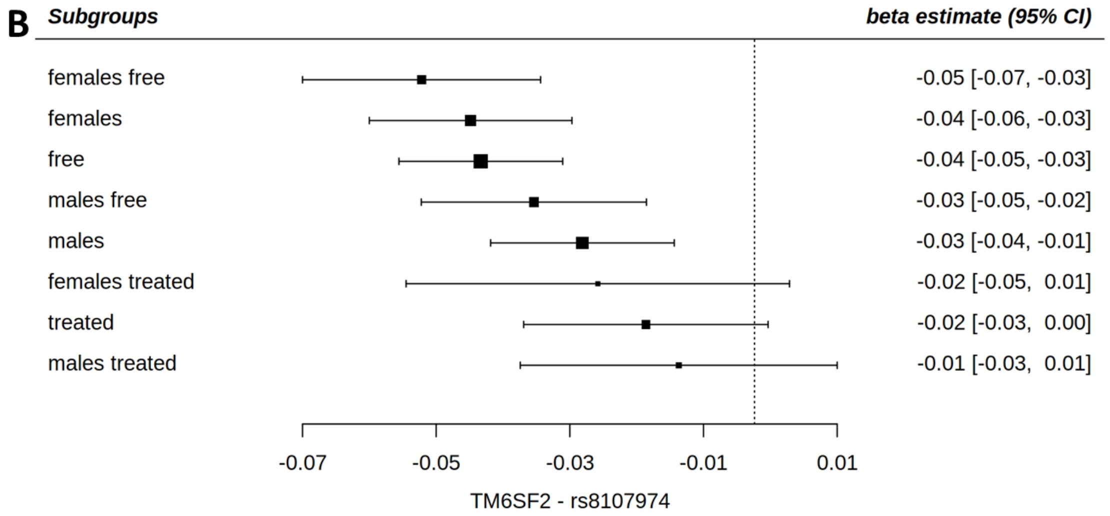

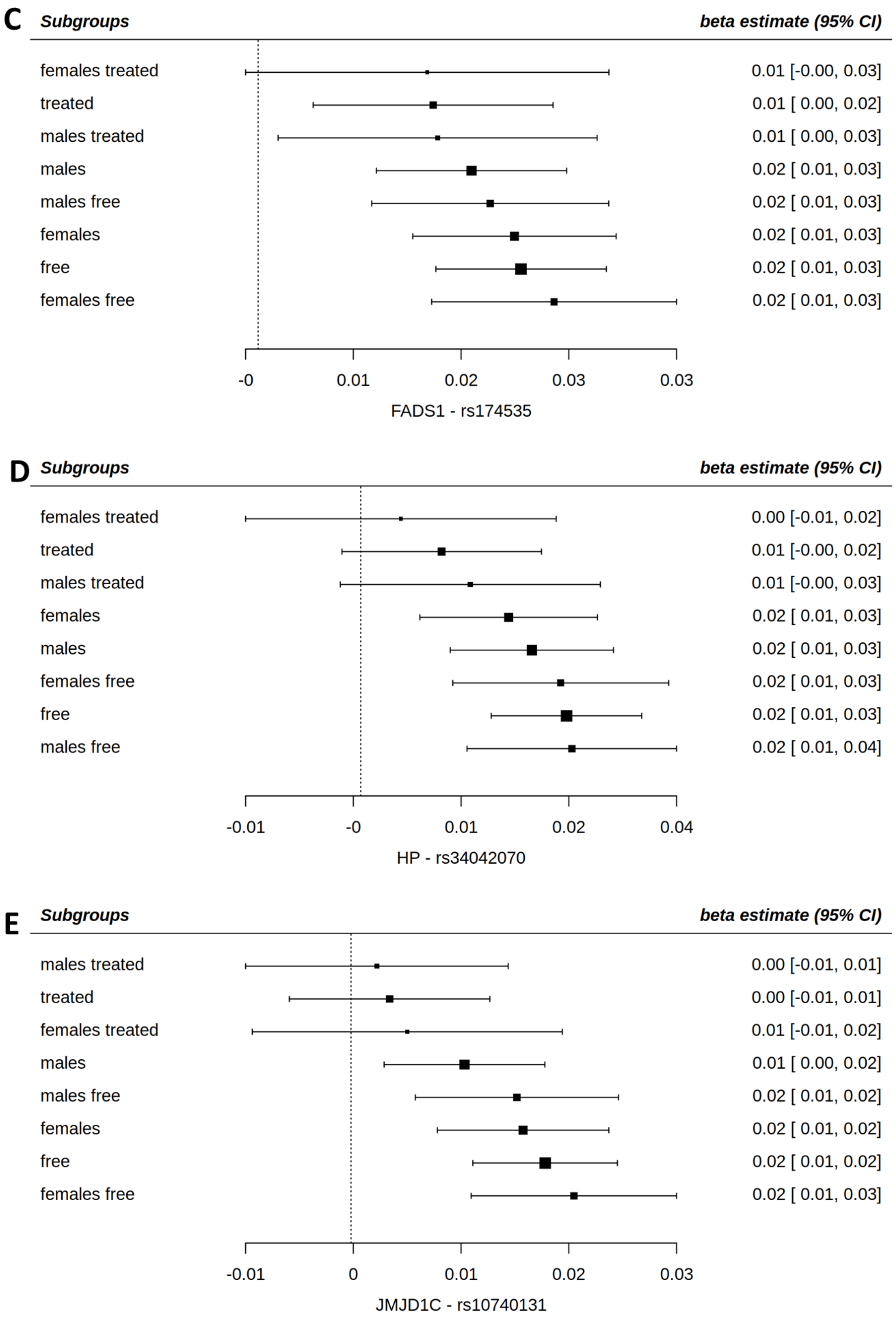
C
Subgroups
beta estimate (95% CI)
females treated 0.01 [-0.00, 0.03]
treated 0.01 [0.00, 0.02]
males treated 0.01 [0.00, 0.03]
males 0.02 [0.01, 0.03]
males free 0.02 [0.01, 0.03]
females 0.02 [0.01, 0.03]
free 0.02 [0.01, 0.03]
females free 0.02 [0.01, 0.03]
-0
0.01
0.02
0.03
0.03
FADS1 - rs174535
D
Subgroups
beta estimate (95% CI)
females treated 0.00 [-0.01, 0.02]
treated 0.01 [-0.00, 0.02]
males treated 0.01 [-0.00, 0.03]
females 0.02 [0.01, 0.03]
males 0.02 [0.01, 0.03]
females free 0.02 [0.01, 0.03]
free 0.02 [0.01, 0.03]
males free 0.02 [0.01, 0.04]
-0.01
-0
0.01
0.02
0.04
HP - rs34042070
E
Subgroups
beta estimate (95% CI)
males treated 0.00 [-0.01, 0.01]
treated 0.00 [-0.01, 0.01]
females treated 0.01 [-0.01, 0.02]
males 0.01 [0.00, 0.02]
males free 0.02 [0.01, 0.02]
females 0.02 [0.01, 0.02]
free 0.02 [0.01, 0.02]
females free 0.02 [0.01, 0.03]
-0.01
0
0.01
0.02
0.03
JMJD1C - rs10740131

Figure S10: Directed acyclic graph (DAG) for Mendelian Randomization (MR). We analyzed the causal effect of PCSK9 on LDL-C, stratified by sex and statin treatment. Statin treatment induces indirectly gene expression of *LDLR* and *PCSK9*. PCSK9 increases the degradation of LDLR and hence increases LDL-C plasma levels. Biological sex is a known risk factor for both PCSK9 and LDL-C.

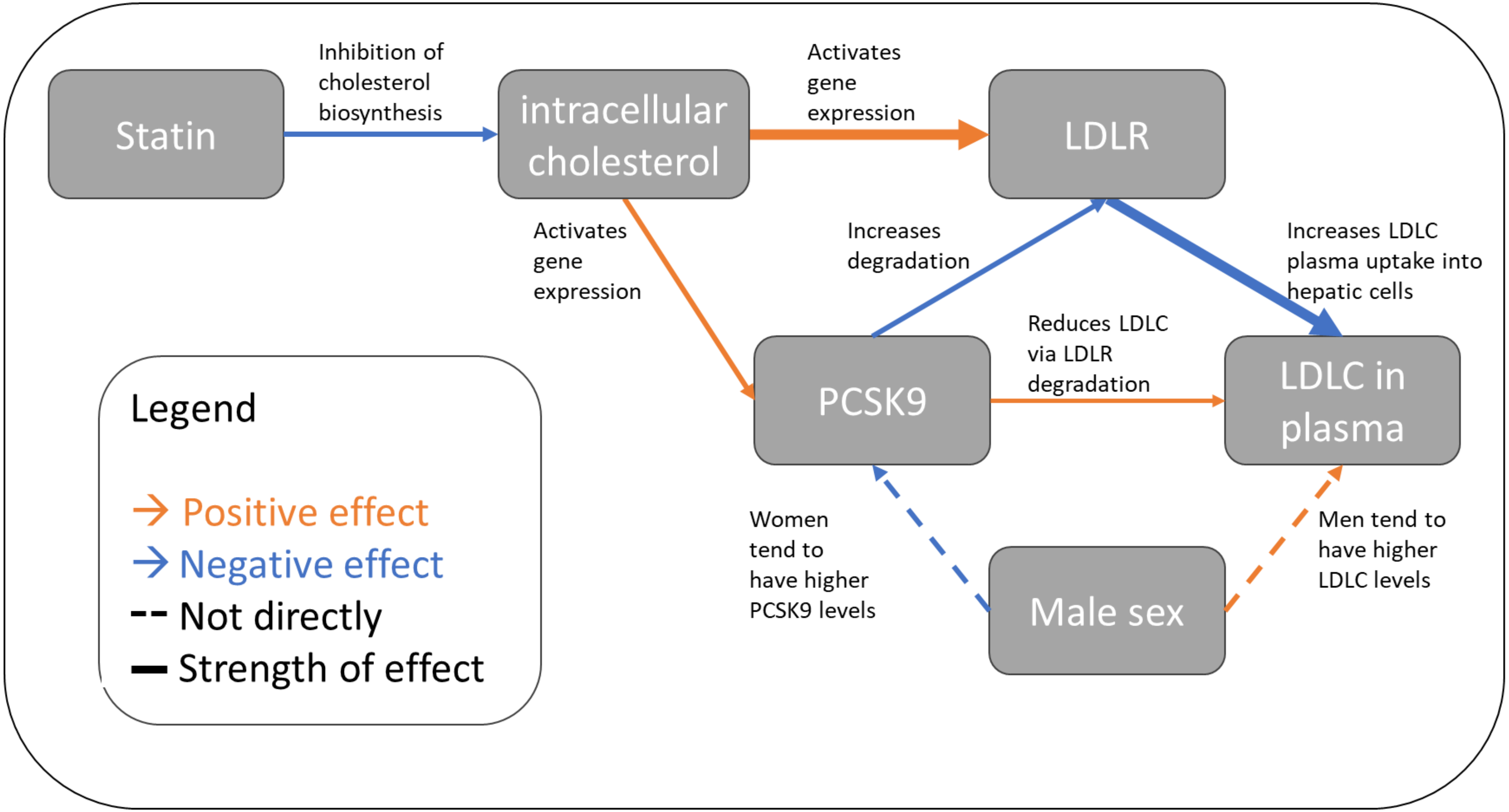

Figure S11: Forest Plot of the causal estimates per subgroup. In each subgroup and SNP, we estimated the Wald ration and used the first term of the delta method for the standard error. Then we combined the single SNP estimates in an inverse-variance-weighted (IVW) meta-analysis (fixed effect), and tested for heterogeneity leaving one SNP out ("w/o SNP x"). Throughout all subgroups, the causal estimate of rs11583680 is weaker than the other three introducing heterogeneity in the IVW analysis. A) Statin-free subjects B) Statin-treated subjects C) Males D) Females E) Statin-free males F) Statin-free females G) Statin-treated males H) Statin-treated females.

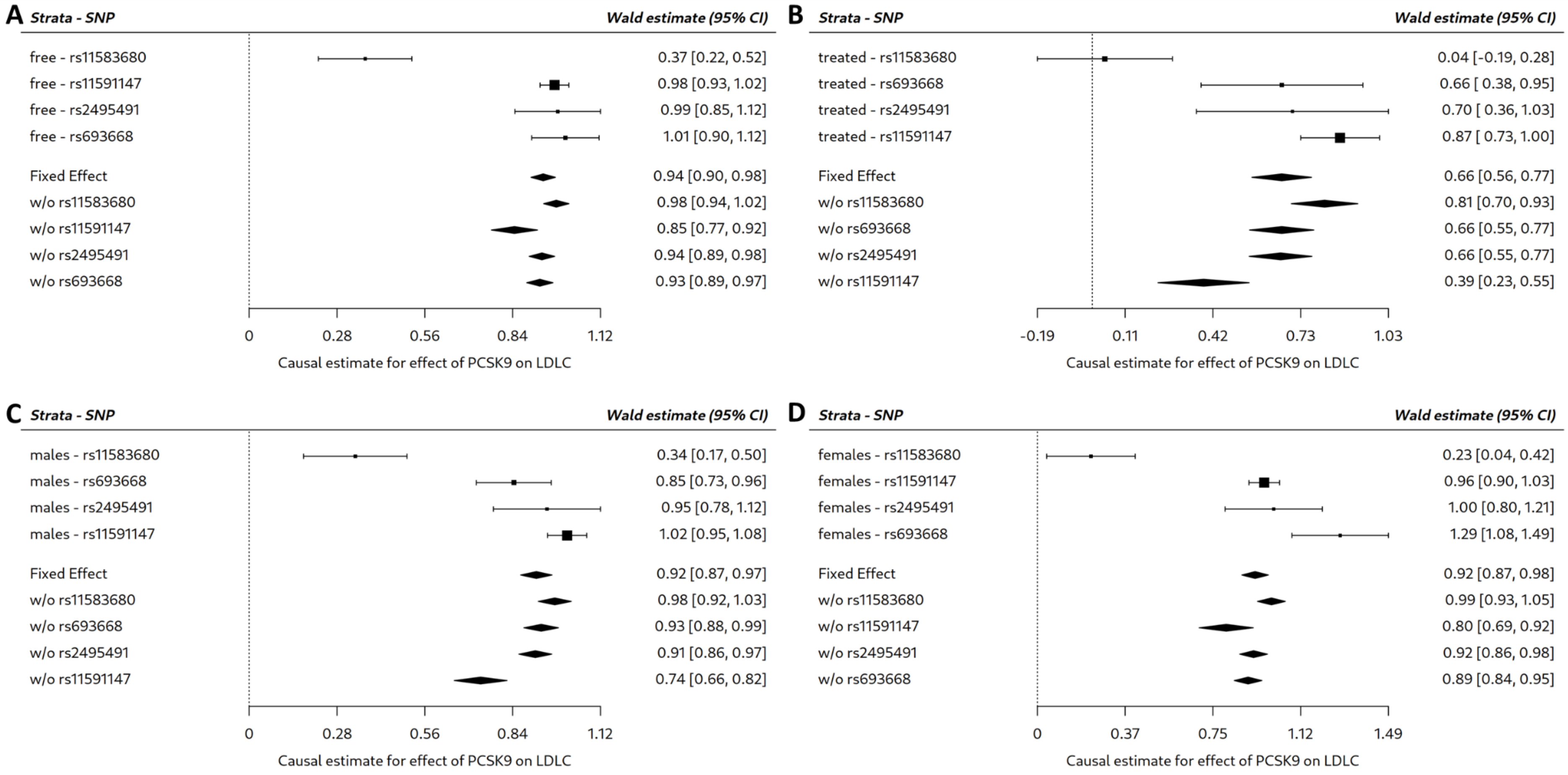

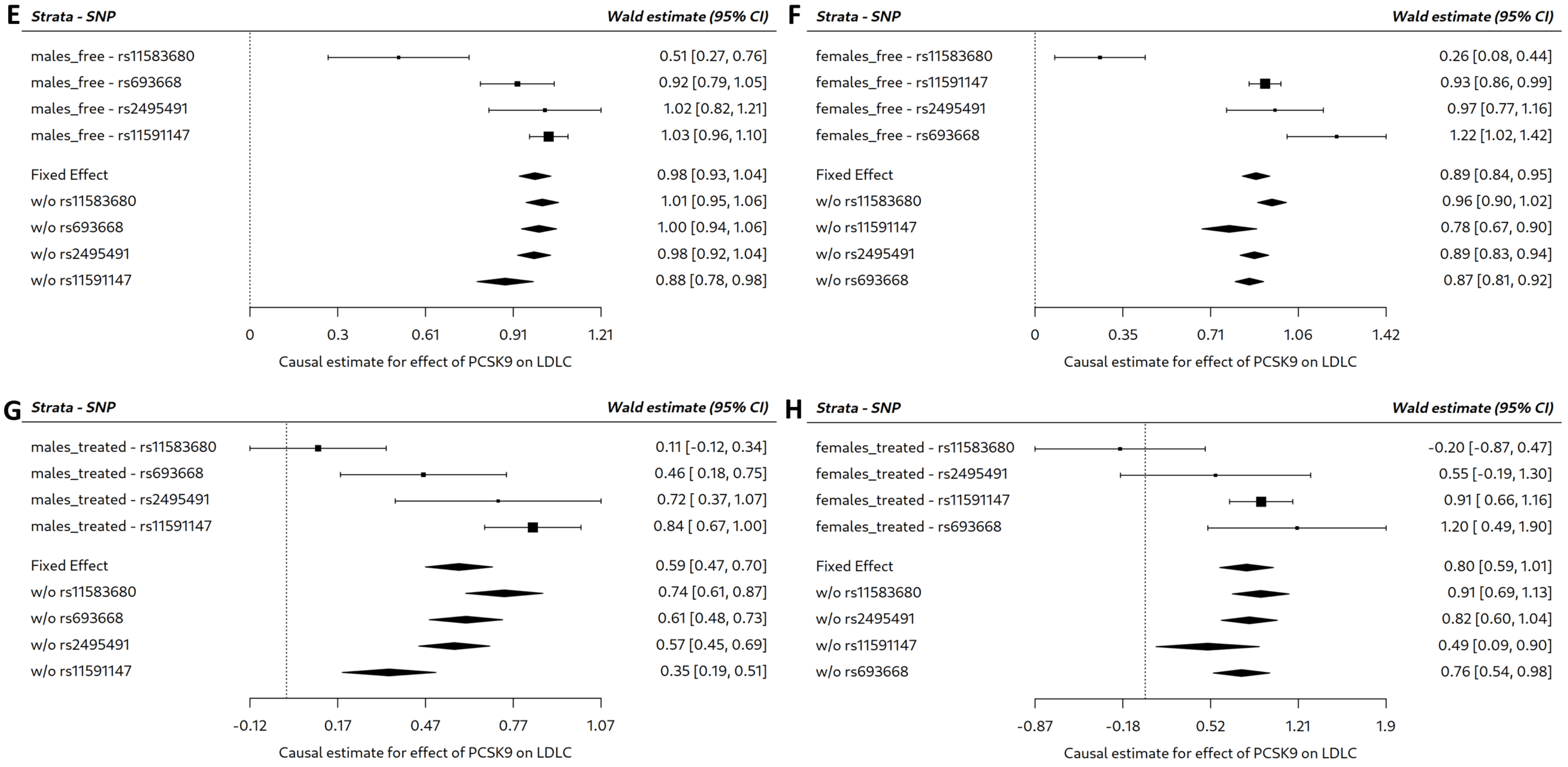
E
Strata - SNP
Wald estimate (95% CI)
males_free - rs11583680
0.51 [0.27, 0.76]
males_free - rs693668
0.92 [0.79, 1.05]
males_free - rs2495491
1.02 [0.82, 1.21]
males_free - rs11591147
1.03 [0.96, 1.10]
Fixed Effect
0.98 [0.93, 1.04]
w/o rs11583680
1.01 [0.95, 1.06]
w/o rs693668
1.00 [0.94, 1.06]
w/o rs2495491
0.98 [0.92, 1.04]
w/o rs11591147
0.88 [0.78, 0.98]
0
0.3
0.61
0.91
1.21
Causal estimate for effect of PCSK9 on LDLC
F
Strata - SNP
Wald estimate (95% CI)
females_free - rs11583680
0.26 [0.08, 0.44]
females_free - rs11591147
0.93 [0.86, 0.99]
females_free - rs2495491
0.97 [0.77, 1.16]
females_free - rs693668
1.22 [1.02, 1.42]
Fixed Effect
0.89 [0.84, 0.95]
w/o rs11583680
0.96 [0.90, 1.02]
w/o rs11591147
0.78 [0.67, 0.90]
w/o rs2495491
0.89 [0.83, 0.94]
w/o rs693668
0.87 [0.81, 0.92]
0
0.35
0.71
1.06
1.42
Causal estimate for effect of PCSK9 on LDLC
G
Strata - SNP
Wald estimate (95% CI)
males_treated - rs11583680
0.11 [-0.12, 0.34]
males_treated - rs693668
0.46 [0.18, 0.75]
males_treated - rs2495491
0.72 [0.37, 1.07]
males_treated - rs11591147
0.84 [0.67, 1.00]
Fixed Effect
0.59 [0.47, 0.70]
w/o rs11583680
0.74 [0.61, 0.87]
w/o rs693668
0.61 [0.48, 0.73]
w/o rs2495491
0.57 [0.45, 0.69]
w/o rs11591147
0.35 [0.19, 0.51]
-0.12
0.17
0.47
0.77
1.07
Causal estimate for effect of PCSK9 on LDLC
H
Strata - SNP
Wald estimate (95% CI)
females_treated - rs11583680
-0.20 [-0.87, 0.47]
females_treated - rs2495491
0.55 [-0.19, 1.30]
females_treated - rs11591147
0.91 [0.66, 1.16]
females_treated - rs693668
1.20 [0.49, 1.90]
Fixed Effect
0.80 [0.59, 1.01]
w/o rs11583680
0.91 [0.69, 1.13]
w/o rs2495491
0.82 [0.60, 1.04]
w/o rs11591147
0.49 [0.09, 0.90]
w/o rs693668
0.76 [0.54, 0.98]
-0.87
-0.18
0.52
1.21
1.9
Causal estimate for effect of PCSK9 on LDLC
